# Efficient Synthesis of Acylated, Dialkyl α-Hydroxy-Benzylphosphonates and Their Anticancer Activity

**DOI:** 10.3390/molecules27072067

**Published:** 2022-03-23

**Authors:** Petra R. Varga, Alexandra Belovics, Péter Bagi, Szilárd Tóth, Gergely Szakács, Szilvia Bősze, Rita Szabó, László Drahos, György Keglevich

**Affiliations:** 1 Department of Organic Chemistry and Technology, Budapest University of Technology and Economics, 1521 Budapest, Hungary; varga.petra.regina@vbk.bme.hu (P.R.V.); alexandra.belovics@gmail.com (A.B.); peter.bagi@gmx.com (P.B.); 2 Research Centre for Natural Sciences, Institute of Enzymology, 1117 Budapest, Hungary; toth.szilard.enzim@ttk.hu (S.T.); gergely.szakacs@meduniwien.ac.at (G.S.); 3 Institute of Cancer Research, Medical University of Vienna, Borschkegasse 8a, A-1090 Vienna, Austria; 4 Eötvös Loránd Research Network (ELKH), Research Group of Peptide Chemistry, Eötvös Loránd University, 1117 Budapest, Hungary; szilvia.bosze@gmail.com (S.B.); rita.szabo@ttk.elte.hu (R.S.); 5 Research Centre for Natural Sciences, MS Proteomics Research Group, 1117 Budapest, Hungary; drahos.laszlo@ttk.hu

**Keywords:** α-hydroxyphosphonates, acylation, triethylamine, cytotoxic activity, anticancer, collateral sensitivity

## Abstract

An efficient method applying acyl chlorides as reagents was developed for the acylation of the hindered hydroxy group of dialkyl α-hydroxy-benzylphosphonates. The procedure did not require any catalyst. A few acylations were also performed with the *S*_C_-enantiomer of dimethyl α-hydroxy-benzylphosphonate, and the optical purity was retained. A part of the acyloxyphosphonates was tested against eight tumor cell lines of different tissue origin at c = 50 μM concentration. The compounds elicited moderate cytostatic effect against breast, skin, prostate, colon, and lung carcinomas; a melanoma cell line; and against Kaposi’s sarcoma cell lines. Then, dose-dependent cytotoxicity was assayed, and benzoylation of the α-hydroxy group was identified as a moiety that increases anticancer cytotoxicity across all cell lines. Surprisingly, a few analogues were more toxic to multidrug resistant cancer cell lines, thus evading P-glycoprotein mediated drug extrusion.

## 1. Introduction

The most important synthesis of α-hydroxyphosphonates is the Pudovik reaction of oxo compounds (aldehydes and ketones) with dialkyl phosphites [1]. Different kinds of base and acid catalysts were described. Base catalyst may be triethylamine [2], TEA/MgCl_2_ [3], barium hydroxide [4,5], or potassium phosphate [6]. Microwave assistance was also useful during the syntheses [7]. A number of solvent-free methods were elaborated for the Pudovik reaction itself; however, the work-up and preparation (extraction, chromatography, recrystallization) requested a considerable quantity of solvent [8,9,10]. One author of this paper together with coworkers developed a green procedure for the synthesis of α-hydroxy-benzylphosphonates. The method comprised the reaction of dialkyl phosphites with benzaldehyde derivatives in a minimum quantity of acetone, in the presence of 5% of triethylamine. After a few hours’ reflux, the adduct precipitated on cooling [11].

Beyond their potential bioactivity, the α-hydroxyphosphonates may be important intermediates in a series of syntheses [1]. The most important reactions are alkylations [1], acylations [12,13,14,15,16,17,18,19,20,21,22,23,24,25,26,27], phosphorylations [28], substitutions [1], rearrangements [1], and dealkylations [1]. As regards acylations, a series of α-(aryloxyacetoxy)-alkylphosphonate derivatives [12,13,14,15] was prepared mainly by acylation of different α-hydroxyphosphonates with carboxylic acid chlorides, including aryloxy-butyryloxy or -valeroxy [16] and heterocyclic derivatives [17,18]. A part of the products prepared were described as herbicidal agents. Among the more complex examples, modification of α-hydroxyphosphonates with acetyl chloride was also described, but in a complicated manner (see below) [19]. Carboxylic acid anhydrides [20,21,22,23] and the acids themselves [24,25,26] were also used in the acylation of hydroxyphosphonates. Among the carboxylic acids, benzoic acid and propionic acid were also applied [25,26]. Comparing the literature examples on the acylation of diethyl α-hydroxy-benzylphosphonates (Table 1), the experiences may be summarized as follows. Using acetyl-chloride in the presence of an iron-doped, single-walled carbon nanotube catalyst at 90 °C without any solvent, the acetyloxy-benzylphosphonate was obtained with a yield of 87% (Table 1, entry 1) [19]. The need for the special catalyst is a disadvantage. Acetic acid anhydride was applied in a solvent-free microwave (MW)-assisted acylation. Although the outcome was practically quantitative (98%), and the reaction time was only 3 min [22], the use of a kitchen MW equipment (and hence, the lack of the temperature range) does not allow a reproduction (Table 1, entry 2). Another method involving Ac_2_O utilized trichlorotitanium trifluoromethanesulfonate as the catalyst at 26 °C in a solvent-free manner affording the product an 89% yield (Table 1, entry 3) [21]. A similar acylation was performed with the anhydride of benzoic acid. In this case, there was a need for 80 °C and for a longer reaction time (Table 1, entry 4) [21]. In both cases, the use of the special catalyst means a disadvantage. Last but not least, α-hydroxy-benzylphosphonate was acylated with propionic acid and benzoic acid under the conditions of the Mitsunobu reaction in boiling acetonitrile, furnishing the corresponding esters in 75/80% yields (Table 1, entries 5 and 6) [26]. Obviously, this is an elegant way of acylation. It is noteworthy that a tosyloxybenzylphosphonate was also described [27]. The phosphorylation of α-hydroxyphosphonates [28] was elaborated by us.

In this paper, we wished to describe a simple and efficient (robust) acylation of α-hydroxyphosphonates. It was also our plan to prepare optically active acylated hydroxyphosphonates and to test the cytotoxic activity of the acyloxy derivatives against different cancer cell cultures.

## 2. Results and Discussion

### 2.1. The Acylation of Racemic Diethyl and Dimethyl α-Hydroxy-Benzylphosphonates

It was shown that acylated α-hydroxyphosphonates were synthesized in different ways applying acyl chlorides, carboxylic acids, and anhydrides under diverse conditions including special catalysts, microwave irradiation, or 4,4′-azopyridine as the reagent of the Mitsunobu reaction. The temperature range embraced 26–90 °C, and the yields fell in the range of 75–90% [19,21,22,26]. As pointed out, none of these methods were too attractive. We wished to use acyl chlorides under simple conditions.

The starting diethyl and dimethyl α-hydroxy-arylmethylphosphonates (**1a**–**d** and **3a**) were prepared as described earlier [11], or by the extension of this method (see Experimental). Derivatives **1c** and **1d** were new.

In the first round, racemic diethyl α-hydroxy-arylmethylphosphonates **1a**–**d** were reacted with 3 equivalents of acetyl chloride in toluene in the presence of 1.1 equivalents of triethylamine. The role of the base was to bind the HCl liberated during acylation. The acylation of unsubstituted **1a** at 25 °C in a closed vessel required a reaction time of 24 h. However, in the other instances with 4-Cl, 4-CF_3_, and 3,4,5-triF electron-withdrawing substituents in the phenyl ring (**1b**, **1c**, and **1d**, respectively), there was need for a higher temperature of 50–60 °C. Then, the diethyl hydroxy-benzylphosphonate (**1a**) was acylated with 1.5 equivalents of butyryl chloride and benzoyl chloride, as described above. After purification by column chromatography, the acyloxyphosphonates (**2a**–**f**) were obtained with yields of 69–97% (Table 2).

Then, racemic dimethyl α-hydroxy-benzylphosphonate (**3a**) was subjected to acylation by reaction with 1.1 equivalents of valeryl-, propionyl-, or benzoyl chloride or 3 equivalents of acetyl chloride in a few combinations. These acylations required more forcing conditions owing to the lower reactivity of the dimethyl esters compared with the diethyl ones (e.g., **3a** vs. **1a**). The C_4_-, C_2_-, and C_1_-acyloxyphosphonates (**4a**–**d**) were prepared in 45–87% yields (Table 3).

All products (**2a**–**f** and **4a**–**d**) were characterized by ^31^P, ^13^C, and ^1^H NMR as well as HRMS. From among the ten acyloxyphosphonates, five (**2c,d** and **4a–c**) were new compounds. ^31^P, ^13^C and ^1^H NMR spectra of the products can be found in the Supplementary Materials section.

The method developed applies acid chlorides in smaller or larger excess to acylate the sterically hindered α-hydroxy group of arylmethylphosphonates (**1** and **3**). Contrary to earlier cases described in the literature, as there was no need for catalysts, and as the yields were mostly high, our method may be regarded a robust one.

### 2.2. The Acylation of the S-Enantiomer of Dimethyl α-Hydroxy-Benzylphosphonate

**Figure 1:**
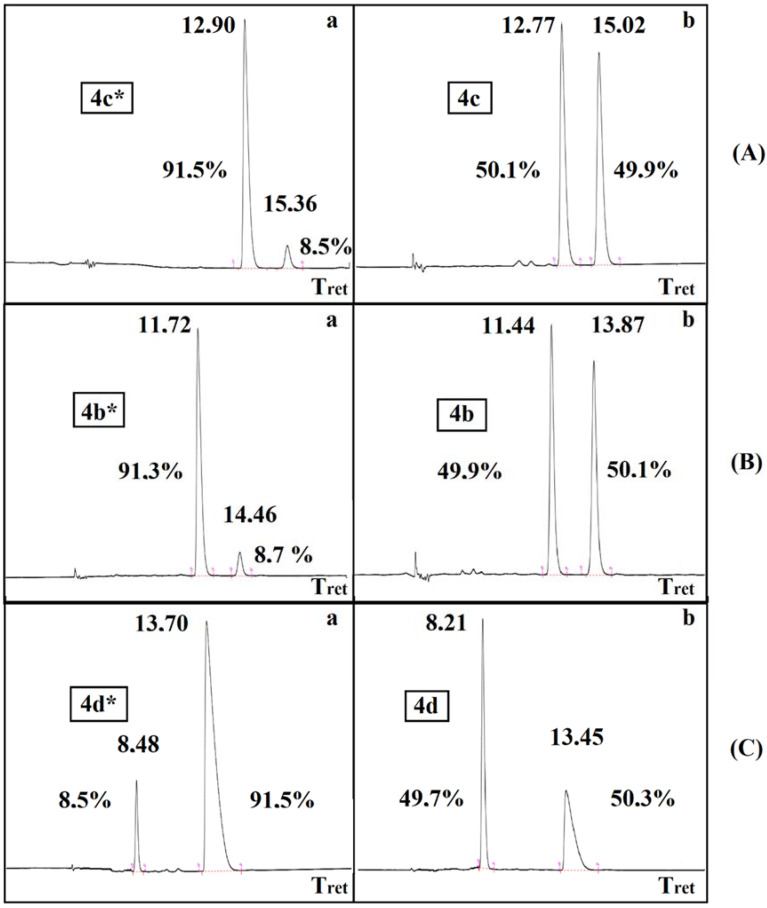
Chiral HPLC chromatograms **Aa**, **Ba**, **Ca** and **Ab**, **Bb**, **Cb** for the optically active 4c*, 4b*, 4d* α-hydroxyphosphonates and for the racemic **4c**, **4b**, **4d** derivatives, respectively.

The resolution of dimethyl α-hydroxy-benzylphosphonate (**3a**) was performed according to an earlier procedure [29]. The optically active *S*-hydroxyphosphonate (3a*) with an *ee* of 84% was also subjected to *O*-acylation with acetyl-, propionyl-, and benzoyl chloride to afford hydroxyphosphonates 4b*, 4c*, 4d*, respectively (Table 4). According to chiral HPLC, no racemization occurred; hence, the optical activity was preserved during the acylations. See Figure 1 (Aa,Ba,Ca). For clarity, the chromatograms of the corresponding racemates are also shown (Figure 1 (Ab,Bb,Cb)). Retention of the configuration is the consequence of the fact that the *O*-acylation does not affect the chirality center. The products were characterized by ^31^P NMR chemical shifts, as well as by specific rotations.

### 2.3. Cytostatic Activity of the Compounds on Various Tumor Cultures with Different Tissue Origin

In vitro cytostatic effect of compounds **1a**, **1c, 1d, 2a, 2c, 2d, 2e**, **2f**, 3a, 4b*, 4c, 4c*, 4d, and 4d* was studied against eight tumor cell lines of different origin (MDA-MB-231 human breast adenocarcinoma [30], A431 human epidermoid carcinoma [31], PC-3 human prostate adenocarcinoma [32], Ebc-1 human lung squamous cell carcinoma [33], MDA-MB-453 human metastatic epithelial breast carcinoma [34], A2058 human melanoma [35], and HT-29 human colon carcinoma [36]) as well as Kaposi’s sarcoma (KS) [37] cells. Cytostatic effect was screened at 50 µM concentration. Cells were treated with the phosphonates overnight; then, the agents were removed by washing and the cells were cultivated for 72 h prior to determining the cell viability.

**Figure 2:**
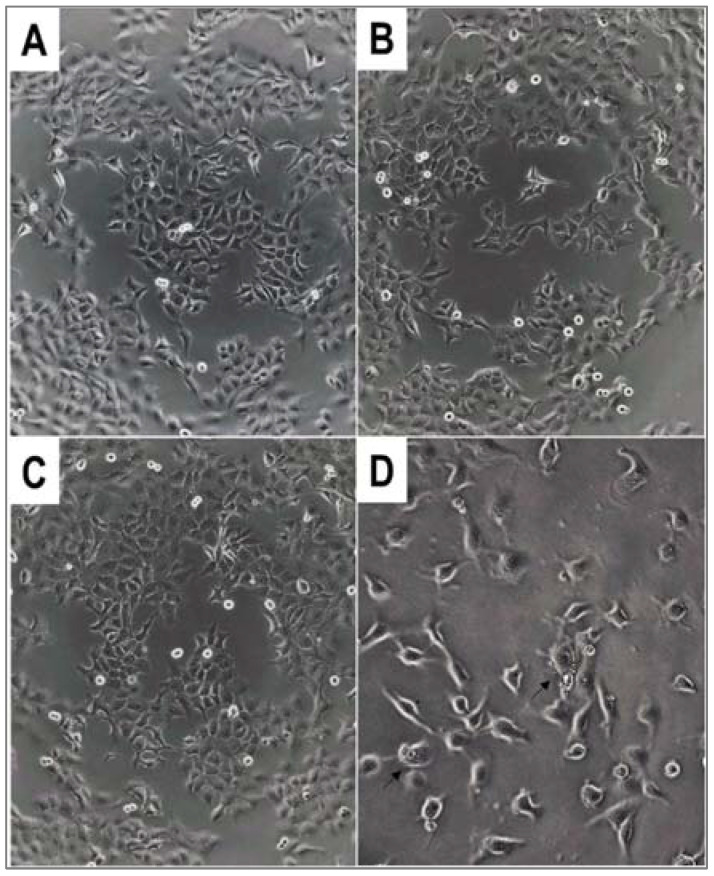
Morphology of A431 human skin carcinoma cells after overnight treatment with α-hydroxyphoshpnate derivatives 4c* and **4d** at c = 50 µM. (**A**) Untreated control (100× magnification); (**B**) 4c* (100×); (**C**) **4d** (100×); (**D**) **4d** (200×). Arrows show membrane blebbing.

The cytostatic effect of the hydroxyphosphonate derivatives was dependent on the cell type; A431 human skin carcinoma and Ebc-1 lung carcinoma proved to be the most sensitive, as several compounds elicited a cytostatic effect against these cell lines, while proliferation of PC-3 prostatic carcinoma and Kaposi’s sarcoma were the least affected by the species studied. The most pronounced cytostatic effect was induced by acylated hydroxyphosphonate **4c** against A2058 human melanoma cells (55.4 ± 1.5%). Derivatives **1a**, **2a**, and **4d** showed significant antitumor effect against A431 cells (40.8 ± 4.8%, 30.5 ± 2.6, and 36.1 ± 0.6%, respectively). Hydroxyphosphonate **1a** and compound **4d** elicited an antiproliferative effect against MDA-MB-231 cells as well (29.7 ± 4,7%, and 36.1 ± 2.3%, respectively). In case of Ebc-1, hydroxyphosphonates **3a** and **2d** proved to be the most effective (36.8 ± 1.9% and 33.0 ± 2.4%, respectively. In case of HT-29 and KS cells, a moderate (<30%) cytostatic effect could be observed. Against HT-29 cells, **1d**, **4c**, 4b*, and 4c* were the most effective compounds, whereas **2c** and **2d** proved to be moderately antiproliferative. The cytostatic activity of the members of the hydroxyphosphonate family is summarized in Table 5 and Figure 2.

The morphology of the cells changed in several cases after the treatment. In a few instances, we could observe the phenomenon of membrane blebbing of the cells, which is characteristic of the early stages of apoptosis (Figure 2D).

### 2.4. Determination of IC_50_ Values

Based on the results shown in Table 5, we decided to measure cytotoxicity to quantify the effect of different substituents. To have a broader view, we chose 7 cell lines of different origin. To investigate and compare the effect of 3,4,5-trifluorination and 3-trifluoromethylation of the benzene ring, and alkylation/arylation of the α-OH group, we assayed the cytotoxicity of phosphonate derivatives **1a**, **1c**,**d**, **2a**, and **2e**,**f** using the PrestoBlue viability reagent. Up to 500 µM, only compound **2f** was toxic enough to obtain IC_50_ values. (It is noted that hydroxyphosphonates **1c,d** were not tested against 143/B.) The toxicity of **2f** was robust throughout the cell panel; IC_50_ values ranged from 234 µM against 143/B osteosarcoma line to 363 µM against CAKI-1 renal carcinoma line, (Table 6) highlighting the contribution of an extra benzene ring to toxicity. Interestingly, when we compared A431 and its ABCB1-expressing multidrug resistant (MDR) derivative, A431-B1, species **2f** was slightly (but not significantly) more toxic to the resistant line.

**Figure 3:**
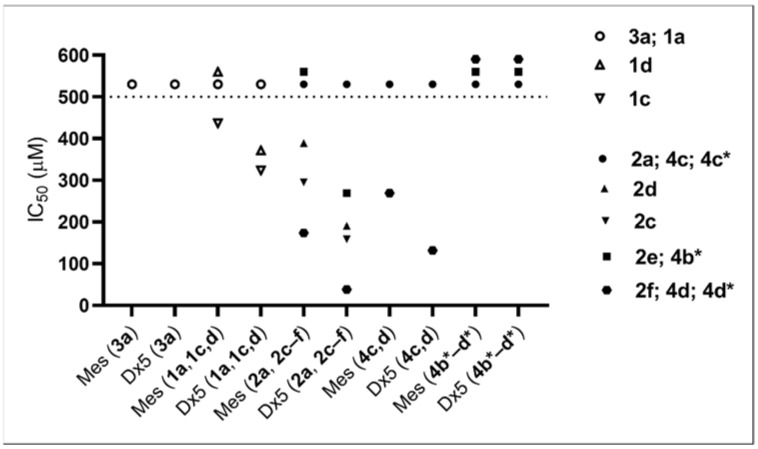
IC_50_ values of the tested compounds against Mes-Sa mCh (Mes) and Mes-Sa/Dx5 eGFP (Dx5). Compounds that did not trigger at least 50% growth inhibition at 500 µM were considered nontoxic; these are shown above the dashed line at 500 µM.

To obtain a more detailed structure–activity relationship, we tested the compounds investigated in the cytostatic screen (see Table 6) against Mes-Sa mCherry (Mes-Sa mCh) and Mes-Sa/Dx5 eGFP (Dx5 eGFP) cells to establish basic SAR for α-hydroxyphosphonates as in an earlier study [38]. The results are shown in Figure 3 and Table 7.

Derivatives **1a** and **3a** of α-hydroxyphosphonates and **2a**, **4c**, and 4c* of α-acylated analogues were not toxic. In our previous study [38], **1a** and **3a** were not toxic against Mes-Sa and Mes-Sa/Dx5 at 200 µM, which is in accordance with our present results. The substitution in the phenyl ring by 3,4,5-trifluorination or 3-trifluoromethylation increased the toxicity. In general, the acylation of the α-hydroxy group resulted in higher toxicity (**1c** vs. **2c**; **1d** vs. **2d**), and a longer alkyl chain (butyl) or a benzyl moiety further increased the toxicity (**2a** < **2e** < **2f**; significant at *p*: 0.05). Compared with methoxy analogue **4d**, benzyl-substituted ethoxy-α-hydroxyphosphonate **2f** was 1.5 times and 3.5 times more toxic against Mes-Sa mCh cell and Dx5 eGFP cell, respectively.

Another important observation is that the enantiomers with *S*-configuration (4b*, 4c*, and 4d*) gave different results compared with their racemic form: none of them killed at least 50% of Mes-Sa mCh or Dx5 eGFP cells up to 500 µM. Thus, the *S*-form seems to be inactive; therefore, most probably the *R*-form is the biologically active entity in the cytotoxicity tests. To clarify this observation, further experiments will be carried out in due course.

The Dx5 eGFP cell line, which is the MDR derivative of Mes-Sa, was more susceptible to the tested compounds. Compound **2f** showed the highest 4.6-fold selectivity to Dx5 eGFP. Due to P-glycoprotein (P-gp) overexpression, Dx5 eGFP is resistant to doxorubicin and other P-gp substrate chemotherapeutics, while, at the same time, hypersensitive to so-called MDR-selective agents, such as NSC57969, which depletes intracellular iron through P-gp, triggering cell death [39,40,41]). To assess if P-gp plays a role in the observed hypersensitivity of Dx5 eGFP cells against the tested analogues, we repeated the experiments in the presence of tariquidar—a P-gp inhibitor (Table 7). One can see a trend in decreasing selectivity that was due to the increased toxicity trend against Mes-Sa mCh and the decreased toxicity trend against Dx5 eGFP. This was a bit unexpected, as mostly, in the presence of tariquidar, the toxicity of compounds against Mes-Sa mCh cells do not change. Phosphonates **1a**, **1c**,**d**, **2a**, **2c**–**f**, **4c**, and 4c*,d* were not toxic to A431 and A431-B1, only species **2f** (Table 6) and **4d** (data not shown) were active with a selectivity of 1.07 and 1.2, respectively. Based on these results, we conclude that increased susceptibility of MDR cells is not conveyed by P-gp. Nevertheless, all of the analogues proved to be equally effective against MDR cells, suggesting that they can evade P-gp-mediated drug resistance in cancer.

## 3. Experimental

### 3.1. General

The ^31^P, ^13^C, ^1^H NMR spectra were taken on a Bruker DRX500 spectrometer (Bruker Corporation, Billerica, MA, USA) operating at 202.4, 125.7, and 500 MHz, respectively. The couplings are given in Hz. HPLC-MS measurements were performed using a Shimadzu LCMS-2020 device equipped with a Reprospher 100 C18 (5 mm; 100 × 3 mm) column and positive–negative double ion source (DUIS) with a quadrupole MS analyzer in a range of 50–1000 *m*/*z*. The sample was eluted with gradient elution using acetonitrile–water 4:1 as the eluent. High-resolution mass spectrometric measurements were performed using a Waters Q-TOF Premier hybrid mass spectrometer in positive electrospray mode (Waters, Manchester, UK). Optical rotations were determined on a Perkin–Elmer 341 polarimeter. The enantiomeric excess (*ee*) values of compounds (4b*, 4c*, 4d*) were determined by chiral HPLC on a Perkin Elmer Series 200 instrument using normal phase mode equipped with Phenomenex Lux^®^ 5 μm Amylose-2 column (250 × 4.6 mm). A mixture of hexane–ethanol was used as the eluent with a flow rate of 0.8 mL/min (T = 20 °C, UV detector α = 254 nm). The conditions and retention times are as follows:

(**4c** and 4c*): hexane/ethanol (85:15), *t*_R1_ 12.8 min (*S*)–**4c**, *t*_R2_ 15.0 min (*R*)–**4c**.

(**4b** and 4b*): hexane/ethanol (85:15), *t*_R1_ 11.4 min (*S*)–**4b**, *t*_R2_ 13.9 min (*R*)–**4b**.

(**4d** and 4d*): hexane/ethanol (50:50), *t*_R1_ 8.2 min (*R*)–**4d**, *t*_R2_ 13.5 min (*S*)–**4d**.

### 3.2. General Procedure for the Synthesis of α-Hydroxyphosphonates (**1a**–**d**, **3a**)

A mixture of 11.0 mmol of substituted aldehyde (benzaldehyde, 1.2 g; 4-chlorobenzaldehyde, 1.5 g; 3-trifluoromethylbenzaldehyde, 1.5 mL; 3,4,5-trifluorobenzaldehyde, 1.2 mL), 11.0 mmol of dialkyl phosphite (dimethyl phosphite, 1.1 mL; diethyl phosphite, 1.4 mL) and 1.1 mmol (0.15 mL) of triethylamine in acetone (1.0 mL) was stirred at reflux for 30 min–6 h (See Table 8). After adding pentane (6.0 mL), the reaction mixture was cooled to 5 °C whereupon the product crystallized from the mixture as a white solid. Filtration afforded products **1a,b,d**, and **3a** in a pure form with yields of 87–95%. In one case, the crude product was purified by column chromatography on silica gel applying dichloromethane–methanol (97:3) as the eluent to afford product (**1c**) as an oil.

#### 3.2.1. Diethyl α-Hydroxy-α-Phenyl-Methylphosphonate (**1a**)

^31^P NMR (CDCl_3_) δ 21.6, δ_P_ (CDCl_3_) 21.7 [11,38]; [M + H]^+^ = 245.

#### 3.2.2. Diethyl α-Hydroxy-α-(4-Chlorophenyl)-Methylphosphonate (**1b**)

^31^P NMR (CDCl_3_) δ 20.8, δ_P_ (CDCl_3_) 21.0 [11,38]; [M + H]^+^ = 278.

#### 3.2.3. Diethyl α-Hydroxy-α-(3-Trifluoromethylphenyl)-Methylphosphonate (**1c**)

^31^P NMR (CDCl_3_) δ 20.5; ^13^C NMR (CDCl_3_) δ 16.3 (d, *J* = 5.9 Hz, OCH_2_*C*H_3_), 63.2 (d, *J* = 7.6 Hz, O*C*H_2_CH_3_), 63.8 (d, *J* = 7.1 Hz, O*C*H_2_CH_3_), 70.1 (d, *J* = 160.2 Hz, P*C*H), 123.9 (dq, *J* = 5.6; 3.7 Hz, C_2_), 124.1 (q, 272.5 Hz, CF_3_), 124.6 (qd/dq, 3.6 Hz, C_4_), 128.5 (d, 2.5 Hz, C_2′_), 130.4 (dq, 5.4, 1.2 Hz, C_5_), 130.5 (qd, 32.1, 2.8 Hz, *C*CF_3_), 138.2 (d, 2.0 Hz, C_1_); ^1^H NMR (CDCl_3_) δ 1.23 (d, *J* = 7.1, 3H, OCH_2_C*H*_3_), 1.26 (d, *J* = 6.9, 3H, OCH_2_C*H*_3_), 4.00–4.13 (m, 2H, OC*H*_2_CH_3_), 5.1 (d, *J* = 10.9, 1H, PC*H*), 7.48 (t, *J* = 7.8, 1H, ArH), 7.57 (d, *J* = 7.9, 1H, ArH), 7.67 (d, *J* = 7.9, 1H, ArH), 7.75–7.78 (m, 1H, ArH); [M + H]^+^ = 313, [M + Na]^+^_found_ = 335.0631, calculated: 335.0636, C_12_H_16_F_3_O_4_PNa.

#### 3.2.4. Diethyl α-Hydroxy-α-(3,4,5-Trifluorophenyl)-Methylphosphonate (**1d**)

^31^P NMR (CDCl_3_) δ 19.8; ^13^C NMR (CDCl_3_) δ 16.13 (d, *J* = 3.4 Hz, OCH_2_*C*H_3_), 16.17 (d, *J* = 3.4 Hz, OCH_2_*C*H_3_), 63.3 (d, *J* = 7.5, O*C*H_2_CH_3_), 63.8 (d, *J* = 6.4, O*C*H_2_CH_3_), 69.1 (d, *J* = 163.3, PCO), 111.0 (dt, *J* = 17.2, C_2_), 133.8 (d, *J* = 8.3, C_1_), 139.03 (dt, *J* = 251.0, 15.5 Hz, C_4_), 148.66–152.84 (m, C_3_); ^1^H NMR (CDCl_3_) 1.28 (t, *J* = 5.9, 3H, OCH_2_C*H*_3_), 1.33 (t, *J* = 5.9, 3H, OCH_2_C*H*_3_), 4.02–4.27 (m, 2H, OC*H*_2_CH_3_), 4.98 (d, *J* = 11.2, 1H, PCH), 7.11–7.22 (m, 2H, ArH); [M + H]^+^ = 299, [M + Na]^+^_found_ = 321.0475, calculated: 321.0480, C_11_H_14_F_3_O_4_PNa.

#### 3.2.5. Dimethyl α-Hydroxy-α-Phenyl-Methylphosphonate (**3a**)

^31^P NMR (CDCl_3_) δ 23.8, δ_P_ (CDCl_3_) 23.8 [11,42]; [M + H]^+^ = 217.

### 3.3. General Procedure for the Synthesis of Acylated Diethyl and Dimethyl α-Hydroxyphosphonates (**2a**–**f**, **4a**–**d**)

To 1.2 mmol of α-hydroxyphosphonate (diethyl hydroxy-benzylphosphonate, 0.28 g; diethyl hydroxy-4-chlorobenzylphosphonate, 0.32 g; diethyl hydroxy-3-trifluoromethyl-benzylphosphonate, 0.36 g; diethyl hydroxy-3,4,5-trifluorobenzylphosphonate, 0.34 g), and 1.3 mmol (0.18 mL) of triethylamine in toluene (4.0 mL), 3.5 mmol (0.25 mL) of acetyl chloride, or 1.7 mmol of other acyl chlorides (butyryl chloride, 0.18 mL; benzoyl chloride, 0.20 mL) were added and the mixture was kept at 25–80 °C for 24 h (See Table 2) in a sealed tube. The precipitated triethylamine hydrochloride was filtered off, and the volatile components were removed in vacuo. The crude product so obtained was purified by column chromatography on silica gel applying dichloromethane–methanol (97:3) as the eluent to give products **2a**–**f** in yields of 69–97% as oils.

To 1.2 mmol (0.25 g) of dimethyl α-hydroxy-benzylphosphonate and 1.2 mmol (0.18 mL) of triethylamine in toluene (4.0 mL) was added to 3.5 mmol (0.25 mL) of acetyl chloride or 1.7 mmol of other acyl chlorides (propionyl chloride, 0.18 mL; butyryl chloride, 0.18 mL; valeryl chloride, 0.18 mL; benzoyl chloride, 0.20 mL) and the mixture was stirred at 25–80 °C for 1–1.5 days (See Table 3). A similar work-up as described above afforded products **4a**–**d** in yields of 45–87%.

The following compounds were thus prepared:

#### 3.3.1. Diethyl α-Acetyloxy-α-Phenyl-Methylphosphonate (**2a**)

^31^P NMR (CDCl_3_) δ 17.7; ^13^C NMR (CDCl_3_) δ 16.23 (d, *J =* 5.6, OCH_2_*C*H_3_), 16.36 (d, *J =* 5.6, OCH_2_*C*H_3_), 20.8 (s, C*C*H_3_), 63.23 (d, *J =* 3.3, O*C*H_2_CH_3_), 63.28 (d, *J =* 4.1, O*C*H_2_CH_3_), 70.4 (d, *J =* 170.1, PCH), 127.9 (d, *J =* 5.9, C_2_^*^), 128.4 (d, *J =* 2.2, C_3_*), 128.7 (d, *J =* 2.9, C_4_), 133.5 (d, *J =* 2.2, C_1_), 169.2 (d, *J =* 8.8, C(O)), *may be reversed; ^1^H NMR (CDCl_3_) δ 1.21 (t, *J =* 7.1, 3H, OCH_2_C*H*_3_), 1.27 (t, *J =* 7.1, 3H, OCH_2_C*H*_3_), 2.2 (s, 3H, C(O)CH_3_), 3.89–4.15 (m, 2H, OC*H*_2_CH_3_), 6.1 (d, *J =* 13.6, 1H, PCH), 7.31–7.40 (m, 3H, ArH), 7.49 (dt, *J =* 8.0, 1.8, 2H); [M + H]^+^ = 287, [M + Na]^+^_found_ = 309.0866, calculated: 309.0868, C_13_H_19_O_5_PNa.

#### 3.3.2. Diethyl α-Acetyloxy-α-(4-Chlorophenyl)-Methylphosphonate (**2b**)

^31^P NMR (CDCl_3_) δ 14.4; ^13^C NMR (CDCl_3_) δ 16.4 (d, *J =* 5.7, OCH_2_*C*H_3_), 16.5 (d, *J =* 5.5, OCH_2_*C*H_3_), 20.9 (s, C*C*H_3_), 63.4 (d, *J =* 7.1, O*C*H_2_CH_3_), 63.5 (d, *J =* 6.4, O*C*H_2_CH_3_), 69.9 (d, *J =* 170.6, PCH), 128.8 (d, *J =* 2.3, C_2_^*^), 129.3 (d, *J =* 5.8, C_3_^*^), 132.2 (d, *J =* 2.4, C_4_), 134.7 (d, *J =* 3.7, C_1_), 169.2 (d, *J =* 8.9, C(O)), *may be reversed; ^1^H NMR (CDCl_3_) δ 1.23 (t, *J =* 7.0, 3H, OCH_2_C*H*_3_), 1.28 (t, *J =* 7.1, 3H, OCH_2_C*H*_3_), 2.2 (s, 3H, C(O)CH_3_), 3.87–4.17 (m, 2H, OC*H*_2_CH_3_), 6.1 (d, *J =* 13.7, 1H, PCH), 7.33–7.36 (m, 2H, ArH), 7.41–7.44 (m, 2H, ArH); [M + H]^+^ = 321, [M + Na]^+^_found_ = 343.0475, calculated: 343.0478, C_13_H_18_ClO_5_PNa.

#### 3.3.3. Diethyl α-Acetyloxy-α-(3-Trifluoromethylphenyl)-Methylphosphonate (**2c**)

^31^P NMR (CDCl_3_) δ 16.7; ^13^C NMR (CDCl_3_) δ 16.1 (d, *J* = 6.8 Hz, OCH_2_*C*H_3_), 16.2 (d, *J* = 6.8 Hz, OCH_2_*C*H_3_), 20.6 (s, C*C*H_3_), 63.35 (d, *J =* 7.1, O*C*H_2_CH_3_), 63.45 (d, *J =* 7.1, O*C*H_2_CH_3_), 69.8 (d, *J =* 169.8, PCH), 123.8 (q, *J =* 272.6, CF_3_), 124.4 (dq *J =* 3.9, C_2_), 125.3 (dq/qd, C_4_), 129.0 (d, *J =* 2.2, C_2_), 130.8 (dq, *J =* 32.6, 2.3, *C*CF3), 131.1 (dq, *J =* 1.2, C_3_), 134.8 (d, *J =* 2.2, C_1_), 169.0 (d, *J =* 8.7, C(O)); ^1^H NMR (CDCl_3_) δ 1.21 (d, *J =* 7.1, 3H, OCH_2_C*H*_3_), 1.26 (d, *J =* 7.1, 3H, OCH_2_C*H*_3_), 2.2 (s, 3H, C(O)C*H*_3_), 3.94–4.17 (m, 2H, OC*H*_2_CH_3_), 6.2 (d, *J =* 13.6, 1H, PCH), 7.48 (t, *J =* 8.0, 2H, ArH), 7.58 (d, *J =* 8.0, 1H, ArH), 7.66 (d, *J*= 7.9, 1H, ArH), 7.71 (s, 1H, ArH); [M + H]^+^ = 355, [M + Na]^+^_found_ = 377.0739, calculated: 377.0742, C_14_H_18_F_3_O_5_PNa.

#### 3.3.4. Diethyl α-Acetyloxy-α-(3,4,5-Trifluorophenyl)-Methylphosphonate (**2d**)

^31^P NMR (CDCl_3_) δ 16.1; ^13^C NMR (CDCl_3_) δ 16.26 (d, *J* = 6.1 Hz, OCH_2_*C*H_3_), 16.34 (d, *J* = 5.9 Hz, OCH_2_*C*H_3_), 20.6 (d, *J* = 2.3 Hz, C*C*H_3_), 63.5 (d, *J* = 6.7 Hz, O*C*H_2_CH_3_), 63.6 (d, *J* = 7.1 Hz, O*C*H_2_CH_3_), 68.9 (d, *J* = 171.2 Hz, PCH), 112.1 (dt, *J* = 17.1, 5.6 Hz, C_2_), 130.0 (d, *J =* 6.8, C_1_), 139.8 (d, *J =* 253.4, C_4_), 150.0 (d, *J =* 10.2, C_3_), 152.0 (d, *J =* 10.0, C_3_), 168.9 (d, *J =* 8.8, *C*(O)); ^1^H NMR (CDCl_3_) δ 1.28 (d, *J =* 7.0, 3H, OCH_2_C*H*_3_), 1.33 (d, *J =* 7.0, 3H, OCH_2_C*H*_3_), 2.2 (s, 3H, C(O)CH_3_), 3.96–4.26 (m, 2H, OC*H*_2_CH_3_), 6.0 (d, *J =* 14.1, 1H, PCH), 7.1 (ArH); [M + H]^+^ = 341, [M + Na]^+^_found_ = 363.0585, calculated: 363.0585, C_13_H_16_F_3_O_5_PNa.

#### 3.3.5. Diethyl α-Butyryloxy-α-Phenyl-Methylphosphonate (**2e**)

**2e**: ^31^P NMR (CDCl_3_) δ 17.9; ^13^C NMR (CDCl_3_) δ 13.7 (s, CH_2_*C*H_3_), 16.4 (d, *J =* 5.9, OCH_2_*C*H_3_), 16.5 (d, *J =* 5.8, OCH_2_*C*H_3_), 18.5 (s, *C*H_2_CH_3_), 36.1 (s, *C*H_2_CH_2_CH_3_), 63.36 (d, *J =* 3.6, O*C*H_2_CH_3_), 63.41 (d, *J =* 4.0, O*C*H_2_CH_3_), 70.2 (d, *J =* 170.0, PCH), 127.9 (d, *J =* 5.8, C_2_^*^), 128.5 (d, *J =* 2.2, C_3_^*^), 128.7 (d, *J =* 3.0, C_4_), 133.6 (d, *J =* 2.2, C_1_), 172.0 (d, *J =* 8.7, *C*(O), *may be reversed; ^1^H NMR (CDCl_3_) δ 1.0 (t, *J =* 7.4, 3H, CH_2_C*H*_3_), 1.21 (t, *J =* 7.1, 3H, OCH_2_C*H*_3_), 1.26 (t, *J =* 7.1, 3H, OCH_2_C*H*_3_), 2.30–2.36 (m, 2H, C(O)CH_2_), 2.48–2.53 (m, 2H, C*H*_2_CH_3_), 3.85–4.17 (m, 4H, OC*H*_2_CH_3_), 6.2 (d, *J =* 13.5, 1H, PCH), 7.32–7.43 (m, 3H, ArH), 7.46–7.50 (m, 2H, ArH); [M + H]^+^ = 315, [M + Na]^+^_found_ = 337.1178, calculated: 337.1181, C_15_H_23_O_5_PNa.

#### 3.3.6. Diethyl α-Benzoyloxy-α-Phenyl-Methylphosphonate (**2f**)

^31^P NMR (CDCl_3_) δ 18.6; ^13^C NMR (CDCl_3_) δ 16.3 (d, *J =* 5.7, OCH_2_*C*H_3_), 16.4 (d, *J =* 5.7, OCH_2_*C*H_3_), 63.5 (d, *J =* 6.7, O*C*H_2_CH_3_), 63.6 (d, *J =* 7.0, O*C*H_2_CH_3_), 70.9 (d, *J =* 170.6, PCH), 127.9 (d, *J =* 5.8, C_2_^*^), 128.5 (d, *J =* 3.9, C_3_^*^), 129.9 (d, *J =* 3.2, C_4_), 133.4 (d, *J =* 2.1, C_1_), 165.0 (d, *J =* 9.1, C(O)), *may be reversed; ^1^H NMR (CDCl_3_) δ 1.20 (t, *J =* 6.9, 3H, OCH_2_C*H*_3_), 1.24 (t, *J =* 7.0, 3H, OCH_2_C*H*_3_), 3.68–4.20 (m, 2H, OC*H*_2_CH_3_), 6.4 (d, *J =* 13.2, PCH), 7.30–7.60 (ArH); [M + H]^+^ = 313, [M + Na]^+^_found_ = 371.1024, calculated: 371.1024, C_18_H_21_O_5_PNa.

#### 3.3.7. Dimethyl α-Valeryloxy-α-Phenyl-Methylphosphonate (**4a**)

^31^P NMR (CDCl_3_) δ 18.8; ^13^C NMR (CDCl_3_) δ 13.6 (CH_2_*C*H_3_), 22.1 (*C*H_2_CH_3_), 26.8 (*C*H_2_CH_2_CH_3_), 33.8 (*C*H_2_C(O)), 53.69 (d, *J =* 6.5, OCH_3_), 53.73 (d, *J =* 7.1, OCH_3_), 69.7 (d, *J =* 169.8, PCH), 127.7 (d, *J =* 5.8, C_2_^*^), 128.5 (d, *J =* 2.2, C_3_^*^), 128.7 (d, *J =* 2.8, C_4_), 133.2 (d, *J =* 2.1, C_1_), 171.9 (d, *J =* 8.4, C(O)), *may be reversed; ^1^H NMR (CDCl_3_) δ 0.91 (t, *J =* 7.3, 3H, CH_2_C*H*_3_), 1.27–1.41 (m, 2H, C*H*_2_CH_3_), 1.55–1.71 (m, 2H, C*H*_2_CH_2_CH_3_), 2.44 (td, *J =* 7.4, *J* = 1.6, 2H, CH_2_C(O)), 3.65 (d, *J* = 10.6, 3H, OCH_3_), 3.72 (d, *J* = 10.7, 3H, OCH_3_), 6.19 (d, *J* = 13.5, 1H, PCH), 7.30–7.42 (m, 3H, ArH), 7.43–7.52 (m, 2H, ArH).

#### 3.3.8. Dimethyl α-Propionyloxy-α-Phenyl-Methylphosphonate (**4b**)

^31^P NMR (CDCl_3_) δ 20.19; ^13^C NMR (CDCl_3_) δ 9.0 (s, CH_2_*C*H_3_), 27.5 (s, *C*H_2_CH_3_), 53.76 (d, *J* = 6.0 Hz, OCH_3_), 53.84 (d, *J* = 6.0 Hz, OCH_3_), 69.9 (d, *J* = 170.0 Hz, PCH), 127.77 (d, *J* = 5.8 Hz, C_2_^*^), 128.6 (d, *J* = 2.2 Hz, C_3_^*^), 128.8 (d, *J* = 2.9 Hz, C_4_), 133.3 (d, *J* = 2.3 Hz, C_1_), 172.7 (dd, *J* = 8.7, Hz, C(O)), *may be reversed; ^1^H NMR (CDCl_3_) δ 1.16 (t, *J* = 7.5 Hz, 3H, CH_2_C*H*_3_), 2.53–2.38 (m, 2H, C*H*_2_CH_3_), 3.64 (d, *J* = 10.5 Hz, 3H, OCH_3_), 3.70 (d, *J* = 10.7 Hz, 3H, OCH_3_), 6.17 (d, *J* = 13.5 Hz, 1H, PCH), 7.30–7.39 (m, 3H, Ar), 7.45–7.49 (m, 2H, ArH).

#### 3.3.9. Dimethyl α-Acetyloxy-α-Phenyl-Methylphosphonate (**4c**)

^31^P NMR (CDCl_3_) δ 20.1; ^13^C NMR (CDCl_3_) δ 20.7 (CH_3_), 53.69 (d, *J =* 6.8, O*C*H_3_), 53.74 (d, *J =* 6.8, O*C*H_3_), 69.9 (d, *J =* 170.0, P*C*H), 127.8 (d, *J =* 5.7, C_2_^*^), 128.5 (d, *J =* 2.6, C_3_^*^), 128.8 (d, *J =* 2.9, C_4_), 133.2 (d, *J =* 2.3, C_1_), 169.1 (d, *J =* 8.8, *C*(O); *may be reversed; ^1^H NMR (CDCl_3_) δ 2.2 (s, 3H, C(O)C*H*_3_), 3.65 (d, *J =* 10.6, 3H, OCH_3_), 3.73 (d, *J =* 10.7, 3H, OCH_3_), 6.2 (d, *J =* 13.5, 1H, PCH), 7.33–7.40 (m, 3H, ArH), 7.48–7.51 (m, 2H, ArH); [M + H]^+^ = 259. [M + Na]^+^_found_ = 281.0555, calculated: 281.0555, C_11_H_15_O_5_PNa.

#### 3.3.10. Dimethyl α-Benzoyloxy-α-Phenyl-Methylphosphonate (**4d**)

^31^P NMR (CDCl_3_) δ 20.26; ^13^C NMR (CDCl_3_) δ 54.0 (d, *J* = 6.5 Hz, OCH_3_), 54.2 (d, *J* = 7.0 Hz, OCH_3_), 70.5 (d, *J* = 170.5 Hz, PCH), 127.9 (d, *J* = 5.8 Hz, C_2_^a^), 128.6 (s, C_2′_^b^), 128.7 (d, *J* = 2.3 Hz, C_3_^a^), 128.96 (d, *J* = 2.9 Hz, C_1_), 129.9 (s, C_3′_^b^), 130.04 (d, *J* = 1.5 Hz, C_4_), 133.2 (d, *J* = 2.2 Hz, C_1′_), 133.6 (s, C_4′_), 164.90 (d, *J* = 8.8 Hz, C(O)), ^a,b^may be reversed; ^1^H NMR (CDCl_3_) δ 3.69 (d, *J* = 10.6 Hz, 3H, OCH_3_), 3.74 (d, *J* = 10.7 Hz, 3H, OCH_3_), 6.4 (d, *J* = 13.3 Hz, 1H, PCH), 7.31–7.41 (m, 2H, Ar), 7.44–7.50 (m, 3H, Ar), 7.56–7.63 (m, 3H, Ar), 8.1 (dd, *J* = 8.0, 1.4 Hz, 2H, ArH).

### 3.4. General Procedure for the Synthesis of Optically Active Acylated Dimethyl α-Hydroxyphosphonates (4b*–4d*)

The (*S*)-acylated hydroxyphosphonates were synthetized according to the procedures for racemic compounds.

To 1.2 mmol (0.25 g) of (*S*)-dimethyl α-hydroxy-benzylphosphonate and 1.2 mmol (0.18 mL) of triethylamine in toluene (4.0 mL) was added 3.5 mmol (0.25 mL) of acetyl chloride or 1.7 mmol of other acyl chlorides (propionyl chloride, 0.18 mL; benzoyl chloride, 0.20 mL) and the mixture was stirred at 25–80 °C for 1–1.5 days (See Table 4). A similar work-up as described above afforded products 4b*–4d* in yields of 85–97%.

4c*: ^31^P NMR (CDCl_3_) δ 20.15; δ_Pracemic_ (CDCl_3_) 18.0 [43]; [M + H]^+^ = 259, [M + Na]^+^_found_ = 281.0553, calculated: 281.0555, C_11_H_15_O_5_PNa; [α]_D_^25^ = −43.3 (*c* = 1.1, CHCl_3_, *ee* = 83%, *S*).

4b*: ^31^P NMR (CDCl_3_) δ 20.18; [M + H]^+^ = 273, [M + Na]^+^_found_ = 295.0712, calculated: 295.0711, C_12_H_17_O_5_PNa; [α]_D_^25^ = −44.0 (*c* = 1.1, CHCl_3_, *ee* = 83%, *S*).

4d*: ^31^P NMR (CDCl_3_) δ 20.23; δ_Pracemic_ (CDCl_3_) 20.1 [44]; [M + H]^+^ = 321, [M + Na]^+^_found_ = 343.0711, calculated: 343.0711, C_16_H_17_O_5_PNa; [α]_D_^25^ = +26.2 (*c* = 1.1, CHCl_3_, *ee* = 83%, *S*).

### 3.5. Cell Lines and Culture Conditions—In Vitro Cytostasis Assays

In vitro cytostatic effect of the compounds was studied on MDA-MB-231 human breast adenocarcinoma [30], A431 human epidermoid carcinoma [31], PC-3 human prostate adenocarcinoma [32], Ebc-1 human lung squamous cell carcinoma [33], MDA-MB-453 human metastatic epithelial breast carcinoma [34], A2058 human melanoma [35], HT-29 human colorectal carcinoma [36], and Kaposi’s sarcoma (KS) [37] cells. MDA-MB 435 cell line was a generous gift of Dr. Angels Fabra, Hospital of Duran e Reynalds, Barcelona, Spain, 1995 and obtained from Dr. Janet E. Price [36]. The other cell lines were generous gifts of Dr. József Tóvári (Department of Experimental Pharmacology, National Institute of Oncology, Budapest, Hungary). MDA-MB-231, MDA-MB-453, PC-3, Ebc-1, and A431 cells were cultured in DMEM medium (Lonza, Basel, Switzerland) supplemented with 10% FBS (EuroClone, Pero, Italy), 2 mM L-glutamine (BioSera, Nuaille, France), penicillin-streptomycin antibiotics mixture (50 IU/mL and 50 μg/mL, respectively), 1 mM sodium pyruvate (both obtained from Lonza, Basel, Switzerland), and 1% nonessential amino acid mixture (BioSera, Nuaille, France). KS, A2058, and HT-29 cells were cultured in RPMI-1640 medium (Lonza, Basel, Switzerland) supplemented with 10% FBS (EuroClone, Pero, Italy), 2 mM L-glutamine (EuroClone, Pero, Italy), and penicillin-streptomycin antibiotics mixture (50 IU/mL and 50 μg/mL, respectively) (Lonza, Basel, Switzerland). The cultures were maintained at 37 °C in a humidified atmosphere with 5% CO_2_. The cells were grown to confluency and then divided into 96-well tissue culture plates (Sarstedt, Nümbrecht, Germany) with the initial cell number of 5.0 × 10^3^ cells/well. After 24 h incubation at 37 °C, the cells were treated with the compounds in 200 μL final volume containing 1.0 *v/v*% DMSO (Merck, Darmstadt, Germany) at 50 μM concentration overnight, whereas control cells were treated with serum-free medium only, or with DMSO (c = 1.0 *v/v*%) at the same conditions. After incubation, the cells were washed twice with serum-free medium. Subsequently, the cells were cultured for additional 72 h in 10% serum containing medium at 37 °C; then, the MTT (Merck, Darmstadt, Germany) solution (at c = 0.37 mg/mL final concentration) was added to each well. The respiratory chain [45] and other electron transport systems [46] reduce MTT, and thereby form non-water-soluble violet formazan crystals within the cell [47]. The amount of these crystals may be determined by spectrophotometry and serves as an estimate for the number of mitochondria, and hence, the number of living cells in the well [48]. After 3 h of incubation with MTT, the cells were centrifuged with 2000 rpm for 5 min and then the supernatant was removed. The obtained formazan crystals were dissolved in DMSO (100 µL) and the optical density (OD) of the samples was measured at λ = 540 nm and 620 nm, respectively, using ELISA Reader (iEMS Reader, Labsystems, Vantaa, Finland). OD_620_ values were subtracted from OD_540_ values. The percent of cytostasis was calculated with the following equation:

Cytostatic effect (%) = [1 − (OD_treated_^/^OD_control_)] × 100

where values OD_treated_ and OD_control_ correspond to the optical densities of the treated and the control wells, respectively. In each case, two independent experiments were carried out with four parallel measurements. Statistical analysis of data was performed using Student’s t test at the 95% confidence level.

### 3.6. Cell Lines and Culture Conditions—In Vitro Cytotoxicity Assays

The 143/B osteosarcoma cell line was a kind gift of Dr. József Balla (University of Debrecen, Hungary). Mes-Sa and Mes-Sa/Dx5 uterine sarcoma cell lines were obtained from ATCC (Manassas, VA, USA) in 2012, where they were characterized by DNA fingerprinting. A431 was obtained from ATCC as well. We purchased CAKI-1, HCT116, MDA-MB-231, and OVCAR-8 from NIH NCI from the NCI-60 cell line panel. 143/B cells were maintained in MEM; A431, Mes-Sa, and Mes-Sa/Dx5 cell lines were maintained in DMEM. NCI-60 panel cell lines were cultivated in RPMI. Media were supplemented with 10% FBS, 5 mmol/L glutamine, and 50 units/mL penicillin and streptomycin. Mes-Sa cells were engineered by lentiviral transduction to stably express the fluorescent protein mCherry, while Mes-Sa/Dx5 cells express eGFP (see more in reference [49]). After thawing, Mes-Sa/Dx5 cells were selected in 500 nM doxorubicin to ensure the overexpression of P-glycoprotein. Cells were periodically tested and resulted negative for mycoplasma contamination with the MycoAlert mycoplasma detection Kit (Lonza, Basel, Switzerland).

A total 2500 cells/well were seeded in 20 µL of the 143/B, A431, and NCI-60 panel cell lines on 384-well plates. Cells settled for 24 h, when serial dilution of the drugs were added in an additional 40 µL. After 72 h incubation, we added PrestoBlue cell viability reagent (Thermo Fisher, Waltham, MA, USA) for an hour in a final volume of 5%. Fluorescent intensity of the reagent was read by a PerkinElmer EnSpire microplate reader (Waltham, MA, USA) at 555 nm excitation and 585 nm emission wavelengths. pIC50 values were calculated by sigmoidal curve fitting by our custom program, written by Judit Sessler in C#.

The fluorescent Mes-Sa mCherry and Mes-Sa/Dx5 eGFP cell lines were seeded and cocultured on 384-well plates with a density of 2 × 1250 cells/well. Cells settled for 24 h when serial dilutions of the drugs were added in an additional 40 µL (with or without tariquidar). After 144 h incubation, the fluorescent intensity of mCherry (585 nm excitation, 615 nm emission) and eGFP (484 nm excitation, 515 nm emission) was measured. pIC50 values were calculated by our custom program. Liquid handling was performed by a Hamilton StarLet robot.

## 4. Conclusions

In summary, a series of hydroxyphosphonates was converted to the corresponding acyloxyphosphonates by the catalyst-free reaction with different acyl chlorides. During the acylation of the *S*_c_-enantiomer, the optical purity was preserved. To evaluate structure–activity relations, the hydroxyphosphonate derivatives were first tested for cytostatic activity at a low concentration range utilizing eight different cell lines. Their activity was cell- and concentration-dependent. Based on the SAR observed, substitution of hydrogens in the phenyl ring, preferably with trifluoromethyl-group, and more preferably in the *meta*- and *ortho*-positions [40], could increase the anticancer cytotoxicity. Attachment of a larger acyl group to the α-OH function also increased the cytotoxicity. This experience may pave future drug development of phosphonates.

## Figures and Tables

**Table 1:** Different ways for the acylation of diethyl-α-hydroxy-benzylphosphonate.

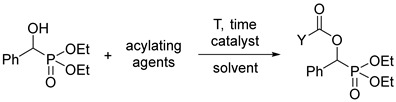								
Entry	Acylating Agents	T (°C)	Time	Catalyst	Solvent	Yield (%)	Remark	Ref.
1	AcCl	90	35 min	Fe-doped single-walled carbon nanotubes	-	87	special catalyst is needed	[21]
2	Ac_2_O	MW/400W ^a^	5 min	-	-	98	not reproducible	[24]
3	Ac_2_O	26	30 min	TiCl_3_(OTf)	-	89	special catalyst is needed	[23]
4	(PhCO)_2_O	80	2	TiCl_3_(OTf)	-	90	special catalyst is needed	[23]
5	CH_3_CH_2_COOH	reflux	15 h	4,4′-azopyridine ^b^	CH_3_CN	75		[28]
6	C_6_H_5_COOH	reflux	12 h	4,4′-azopyridine ^b^	CH_3_CN	80		[28]

^a^ No exact temperature was provided due to the use of a kitchen oven. ^b^ Was applied in equivalent quantity as a reagent.

**Table 2:** The acylation of diethyl α-hydroxy-arylmethylphosphonates **1a**–**d**.

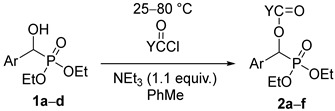							
Entry	Starting Material	Product	Ar	Y	T (°C)	Time (h)	Yield (%)
1	**1a**	**2a**	Ph	Me	25	24	86
2	**1b**	**2b**	4-ClPh	Me	50	24	84
3	**1c**	**2c**	3-CF_3_Ph	Me	60	24	69
4	**1d**	**2d**	3,4,5-triFPh	Me	50	24	77
5	**1a**	**2e**	Ph	Pr	60	24	97
6	**1a**	**2f**	Ph	Ph	80	24	88

**Table 3:** The acylation of dimethyl α-hydroxy-arylmethylphosphonates (**3a**).

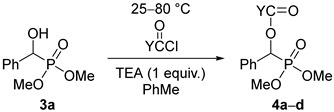					
Entry	Product	Y	T (°C)	Time (h)	Yield (%)
1	**4a**	Bu	80	24	58
2	**4b**	Et	80	24	87
3	**4c**	Me	25	36	80
4	**4d**	Ph	80	24	80

**Table 4:** The acylation of optically active dimethyl α-hydroxy-benzylphosphonate 3a*.

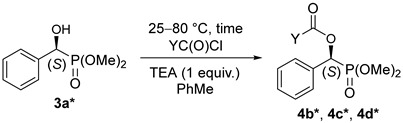						
Entry	Starting Material	Product	Y	T (°C)	Time (h)	Yield (%)
1	3a*	4c*	Me	25	36	97
2		4b*	Et	80	24	95
3		4d*	Ph	80	24	85

**Table 5:** Cytostatic effect ^$^ of α-hydroxy-benzylphosphonate derivatives against human tumor cell lines.

Cell line ^§^	Hydroxyphosphonate Derivatives													
	3a	1a	1c	1d	2a	2c	2d	2e	2f	4c	4d	4b*	4c*	4d*
MDA-MB 231														
MDA-MB 453														
A2058														
A431														
PC-3														
Ebc-1														
HT-29														
KS					.					.				

^§^ Cytostatic effect is color coded as follows: light yellow, cytostasis >10%; yellow, cytostasis >20%; orange boxes, cytostasis >30%; red, cytostasis >50%. Statistical analysis was performed by Student’ *t*-test. Colored fields represent significance at the 5% level (*p* ≤ 0.05).

**Table 6:** Cytotoxicity of hydroxyphosphonate derivative **2f** and control compounds against the panel of cell lines used. SD: standard deviation of IC_50_ values.

	2f		Cisplatin		Doxorubicin		NSC57969	
	IC_50_ [µM]	SD	IC_50_ [µM]	SD	IC_50_ [µM]	SD	IC_50_ [µM]	SD
134/B	234.4	+36.0	3.02	+0.47	0.11	+0.04	3.81	+0.88
		−31.2		−0.41		−0.03		−0.71
CAKI-1	363.1	+90.9	4.57	+1.20	0.15	+0.07	-	
		−72.7		−0.95		−0.05		
HCT 116	257.0	+12.7	4.18	+1.13	0.19	+0.03	-	
		−12.1		−0.89		−0.02		
MDA-MB-231	323.6	+20.0	17.38	+1.85	0.22	+0.09	-	
		−18.8		−1.67		−0.07		
OCVAR-8	316.2	+33.7	8.61	+1.81	0.21	+0.09	-	
		−30.5		−1.50		−0.06		
A431	302.0	+9.89	-		0.14	+0.03	4.36	+1.66
		−9.58				−0.03		−1.20
A431-B1	281.8	+43.2	-		3.17	+0.76	1.12	+0.46
		−37.5				−0.61		−0.32

**Table 7:** Cytotoxicity expressed as IC_50_ values and standard deviation (SD) of phosphonate analogues. TQ: presence of 1 µM tariquidar. nt: no IC_50_ value was detected up to 500 µM. *p* < 0.05, *; *p* < 0.01,**.

	Mes-Sa mCh		Mes-Sa mCh (TQ)		Dx5 eGFP		Dx5 eGFP (TQ)		SR	SR (TQ)
	IC50 [µM]	SD	IC50 [µM]	SD	IC50 [µM]	SD	IC50 [µM]	SD		
**1a**	nt		-		nt		-		-	-
**1c**	407	+76	389	+75	331	+30	437	+54	1.2	0.9
		−64		−63		−28		−48		
**1d**	nt		nt		389	+77	nt		>1.3	-
						−64				
**2a**	nt		-		nt		-		-	-
**2c**	302	+38	257	+22	178	+41	214	+41	1.7 *	1.2
		−34		−20		−33		−35		
**2d**	413	+85	380	+68	219	+63	295	+28	1.9 *	1.3
		−70		−57		−49		−25		
**2e**	nt		465	+68	269	+13	234	+72	>1.9 **	2.0 *
				−59		−12		−55		
**2f**	174	+16	123	+15	38	+5	59	+19	4.6 **	2.1 **
		−15		−14		−5		−14		
**3a**	nt		-		nt		-		-	-
**4c**	nt		-		nt		-		-	-
**4d**	269	+38	236	+39	132	+14	214	+54	2.0 **	1.1
		−33		−33		−13		−43		
4b*	nt		-		nt		-		-	-
4c*	nt		-		nt		-		-	-
4d*	nt		-d		nt		-		-	-
Cisplatin	1.77	+0.69	-		2.34	+0.47	-		0.8	-
		−0.5				−0.39				
Doxorubicin	0.040	+0.02	0.026	+0.01	2.610	+1.59	0.024	+0.02	0.015 **	1.1
		−0.01		−0.01		−0.99		−0.01		
NSC57969	3.87	+1.16	3.48	+0.82	0.51	+0.15	2.01	+0.54	7.5 **	1.7 *
		−0.89		−0.66		−0.12		−0.43		

**Table 8:** Synthesis of α-hydroxyphosphonates [13].

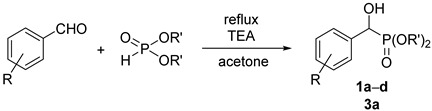							
Entry	Product	R	R’	t (h)	Yield (%)	Workup	Product
1	**1a**	H	Et	2.5	95	pentane, crystallization	white solid
2	**1b**	4-Cl	Et	1	87	pentane, crystallization	white solid
3	**1c**	3-CF_3_	Et	3	75	column chromatography	oil
4	**1d**	3,4,5-triF	Et	3	90	pentane, crystallization	white solid
5	**3a**	H	Me	2.5	95	pentane, crystallization	white solid

## Data Availability

Not relevant.

## Supplementary Materials

The following are available online at https://www.mdpi.com/article/10.3390/molecules27072067/s1, copies of the ^31^P, ^13^C, and ^1^H NMR spectra.

## Sample Availability

Samples of the compounds are available from the authors.
