# Supplementary material for: Efficient Synthesis of Acylated, Dialkyl α-Hydroxy-Benzylphosphonates and Their Anticancer Activity
Source: Molecules. 2022 Mar 23;27(7):2067. doi: 10.3390/molecules27072067 (PMC9000670; doi:10.3390/molecules27072067)
Supplement: Supplementary file 1 [file molecules-27-02067-s001.zip › molecules-1652209-supplementary.pdf]

Supplementary Information

Efficient synthesis of acylated, dialkyl α-hydroxy-benzylphosphonates and their anticancer activity

Petra R. Varga[1], Alexandra Belovics[1], Péter Bagi[1], Szilárd Tóth[2], Gergely Szakács[3], Szilvia Bősze[4], Rita Szabó[4], László Drahos[5], György Keglevich[1,*]

[1]*Department of Organic Chemistry and Technology, Budapest University of Technology and Economics, 1521 Budapest, Hungary*

[2]*Research Centre for Natural Sciences, Institute of Enzymology, 1117 Budapest, Hungary*

[3]*Institute of Cancer Research, Medical University of Vienna, Borschkegasse 8a, A-1090 Vienna, Austria*

[4]*Eötvös Loránd Research Network (ELKH), Research Group of Peptide Chemistry, Eötvös Lorand University, 1117 Budapest, Hungary*

[5]*Research Centre for Natural Sciences, MS Proteomics Research Group, 1117 Budapest, Hungary*

EXPERIMENTAL

All chemicals were purchased from Sigma-Aldrich.

^{31}P, ^{13}C and ^{1}H NMR spectra

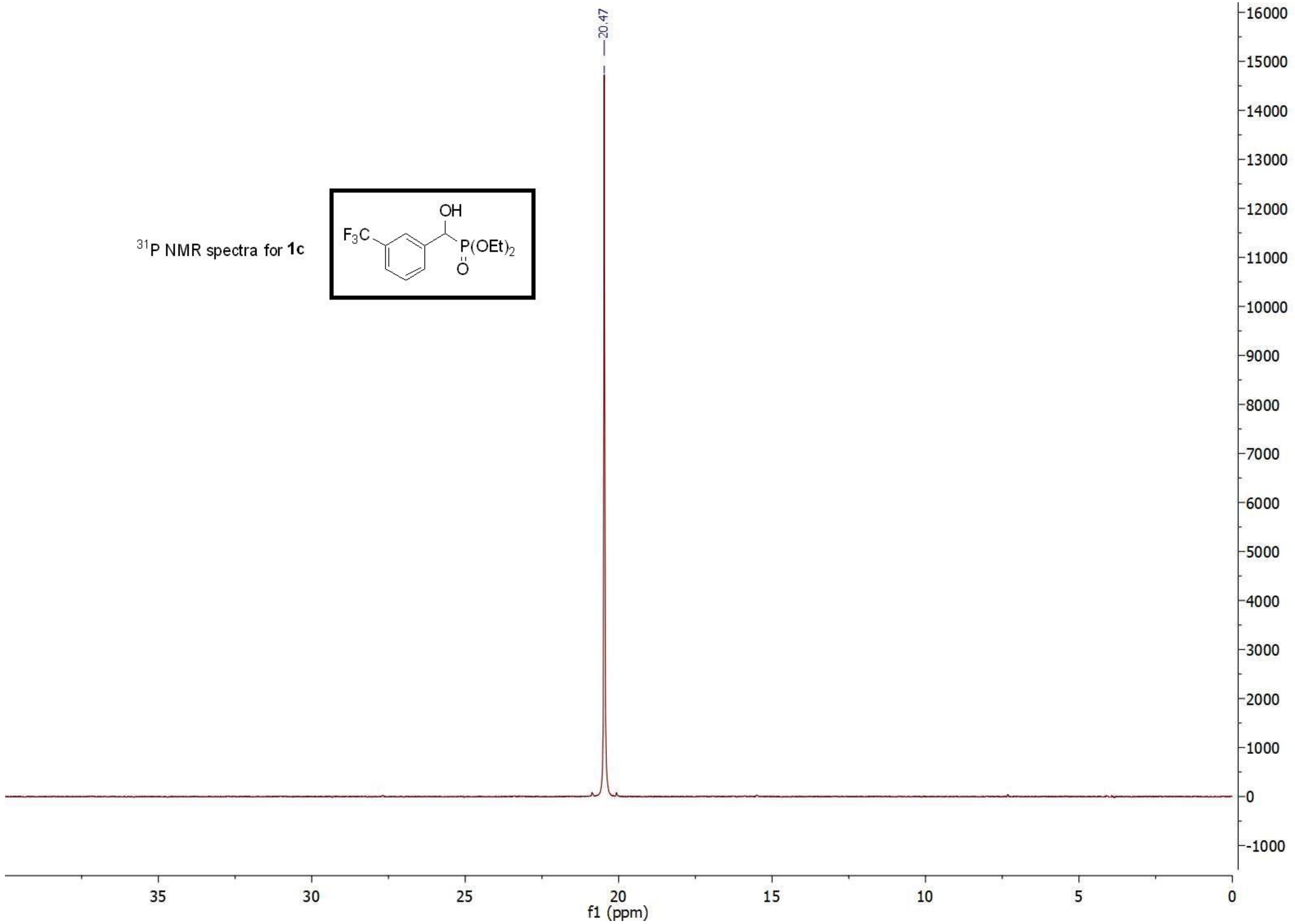

31P NMR spectra for 1c
OH
F3C
P(OEt)2
O
20.47
f1 (ppm)

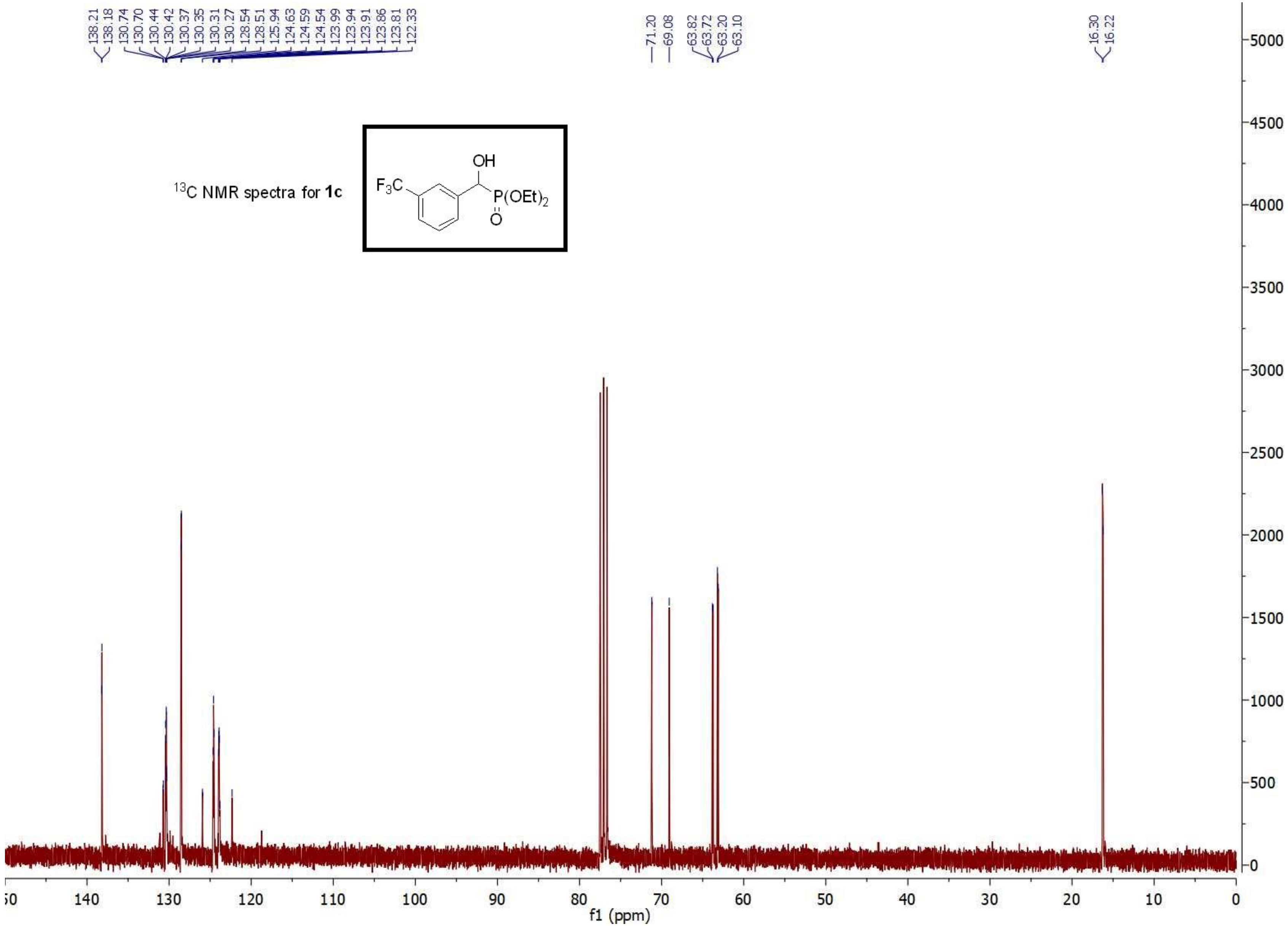
^{13}C NMR spectra for **1c**
OH
F_3C
$P(OEt)_2$
O
138.21
138.18
130.74
130.70
130.44
130.42
130.37
130.35
130.31
130.27
128.54
128.51
125.94
124.63
124.59
124.54
123.99
123.94
123.91
123.86
123.81
122.33
71.20
69.08
63.82
63.72
63.20
63.10
16.30
16.22
f1 (ppm)

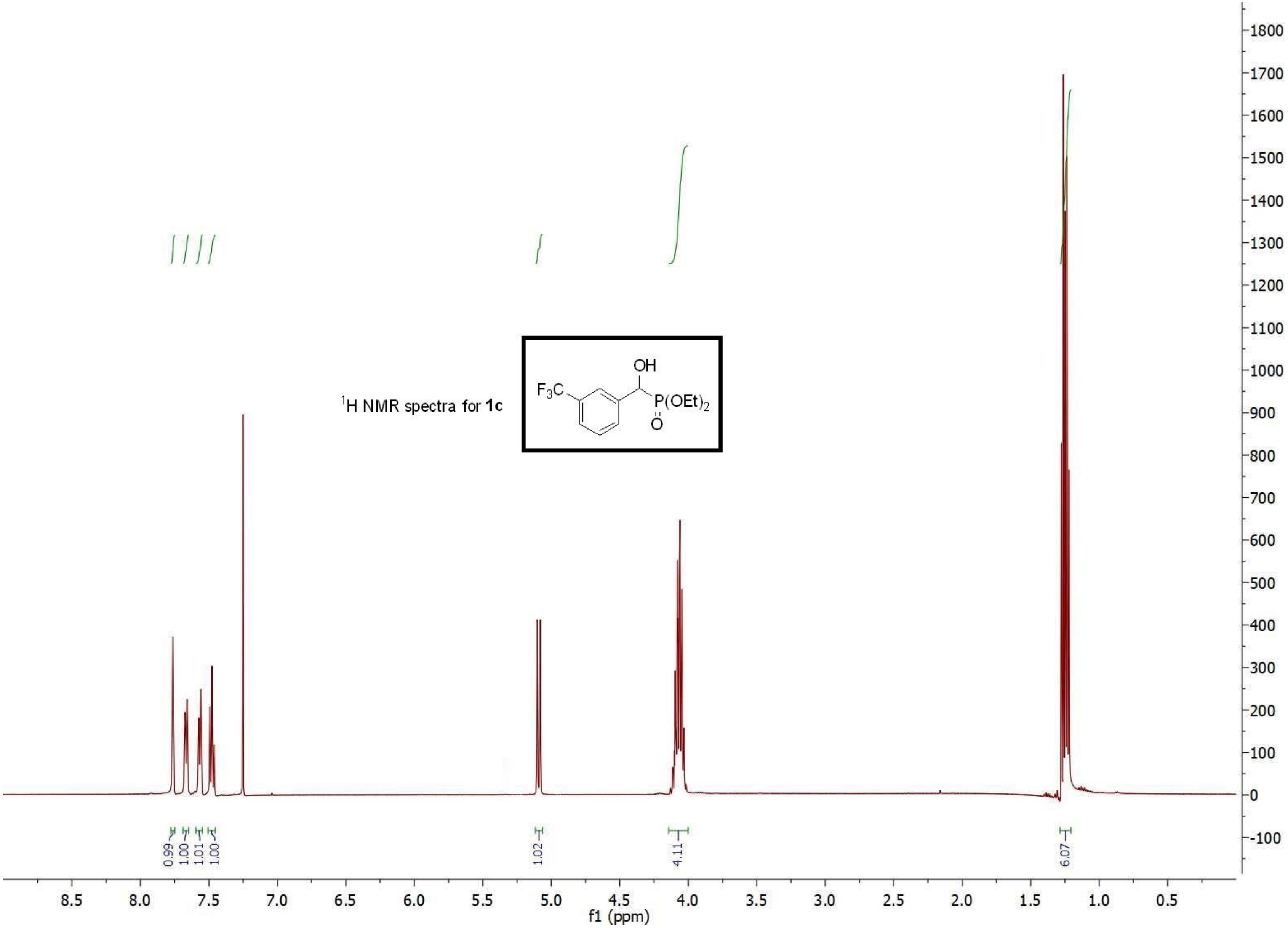

1H NMR spectra for 1c
OH
F3C
P(OEt)2
O
0.99
1.00
1.01
1.00
1.02
4.11
6.07
8.5
8.0
7.5
7.0
6.5
6.0
5.5
5.0
4.5
4.0
3.5
3.0
2.5
2.0
1.5
1.0
0.5
f1 (ppm)
1800
1700
1600
1500
1400
1300
1200
1100
1000
900
800
700
600
500
400
300
200
100
0
-100

^{31}P NMR spectra for **1d**

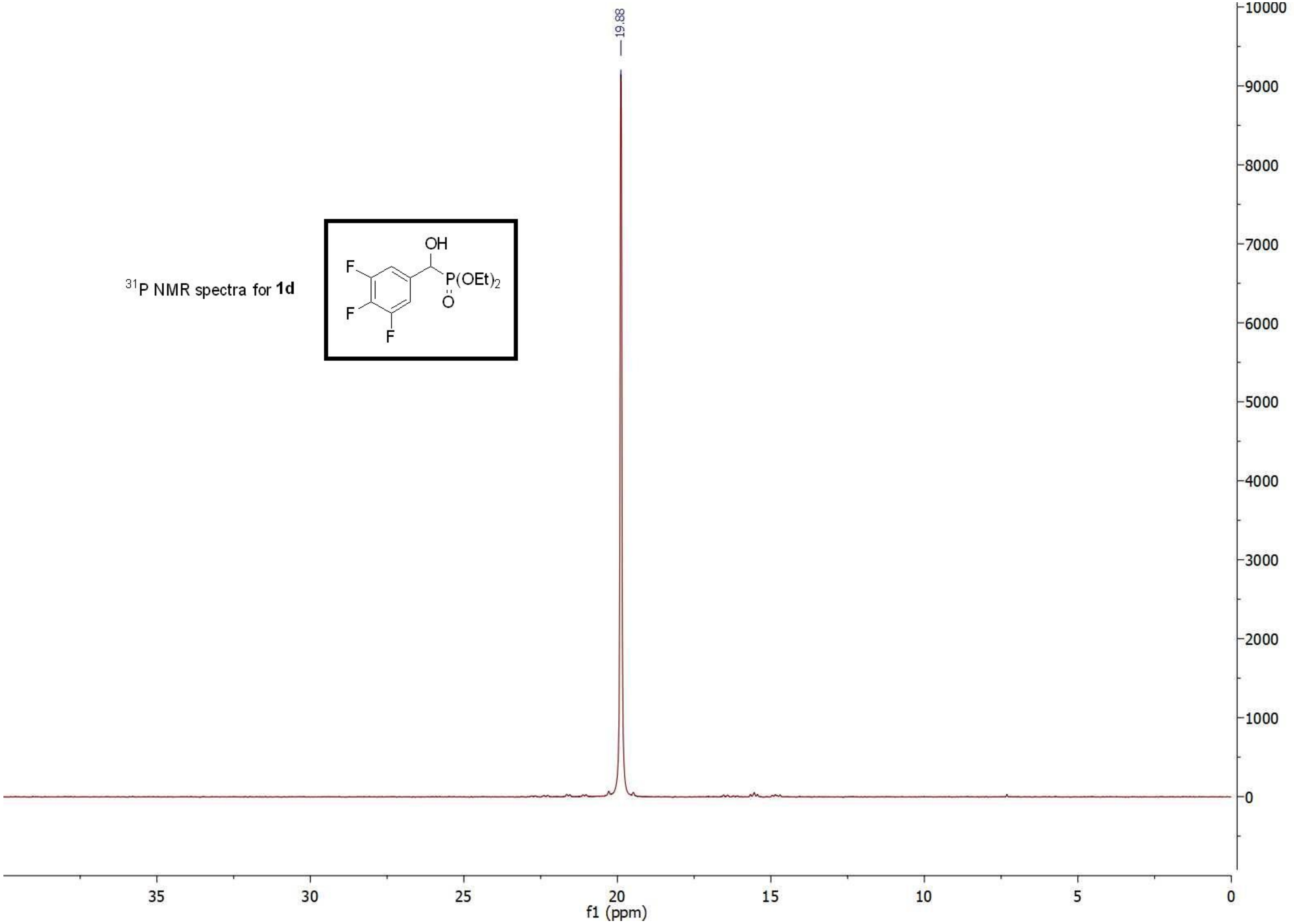

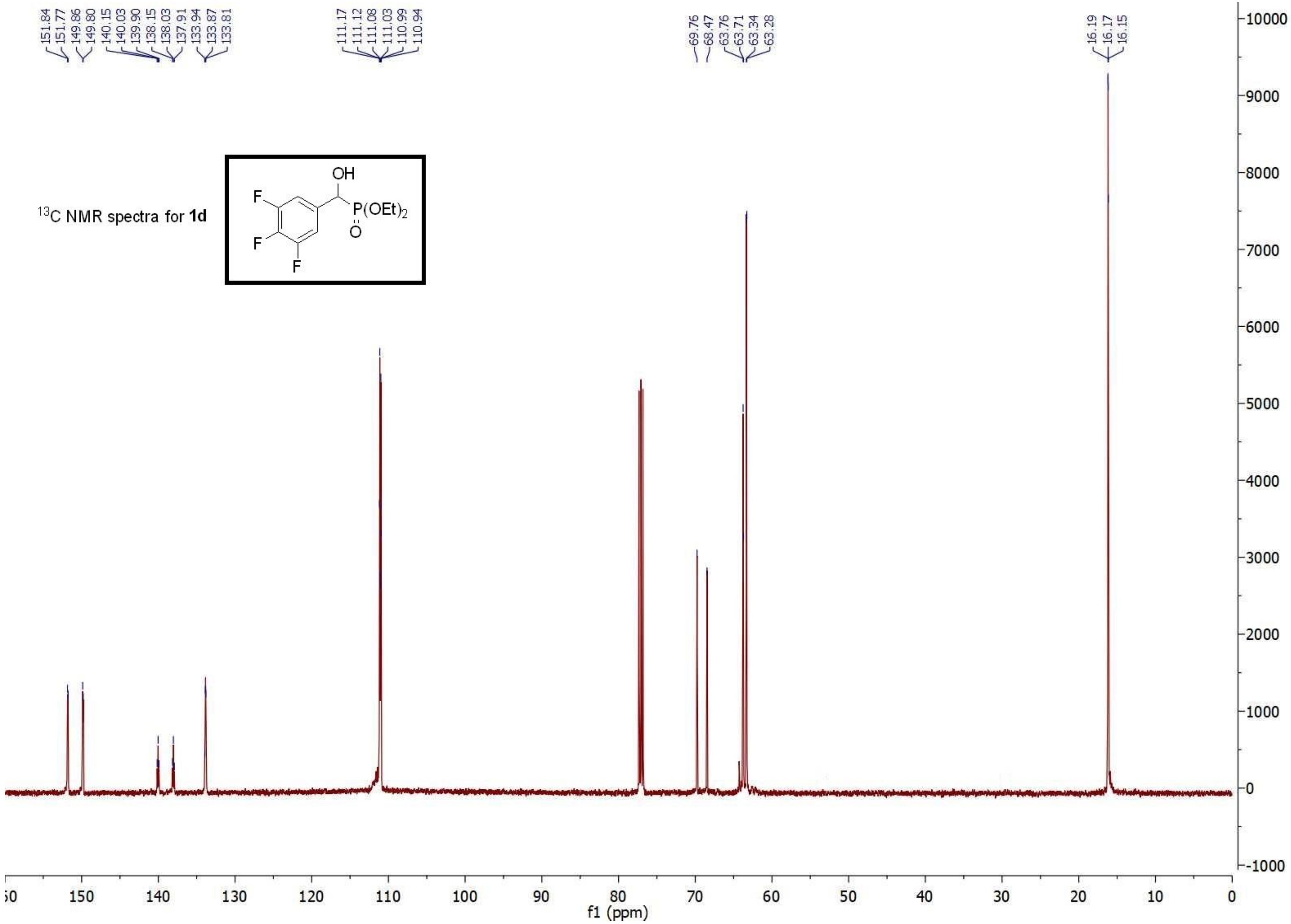

^{13}C NMR spectra for **1d**
OH
F
F
F
P(OEt)$_2$
O
151.84
151.77
149.86
149.80
140.15
140.03
139.90
138.15
138.03
137.91
133.94
133.87
133.81
111.17
111.12
111.08
111.03
110.99
110.94
69.76
68.47
63.76
63.71
63.34
63.28
16.19
16.17
16.15
f1 (ppm)

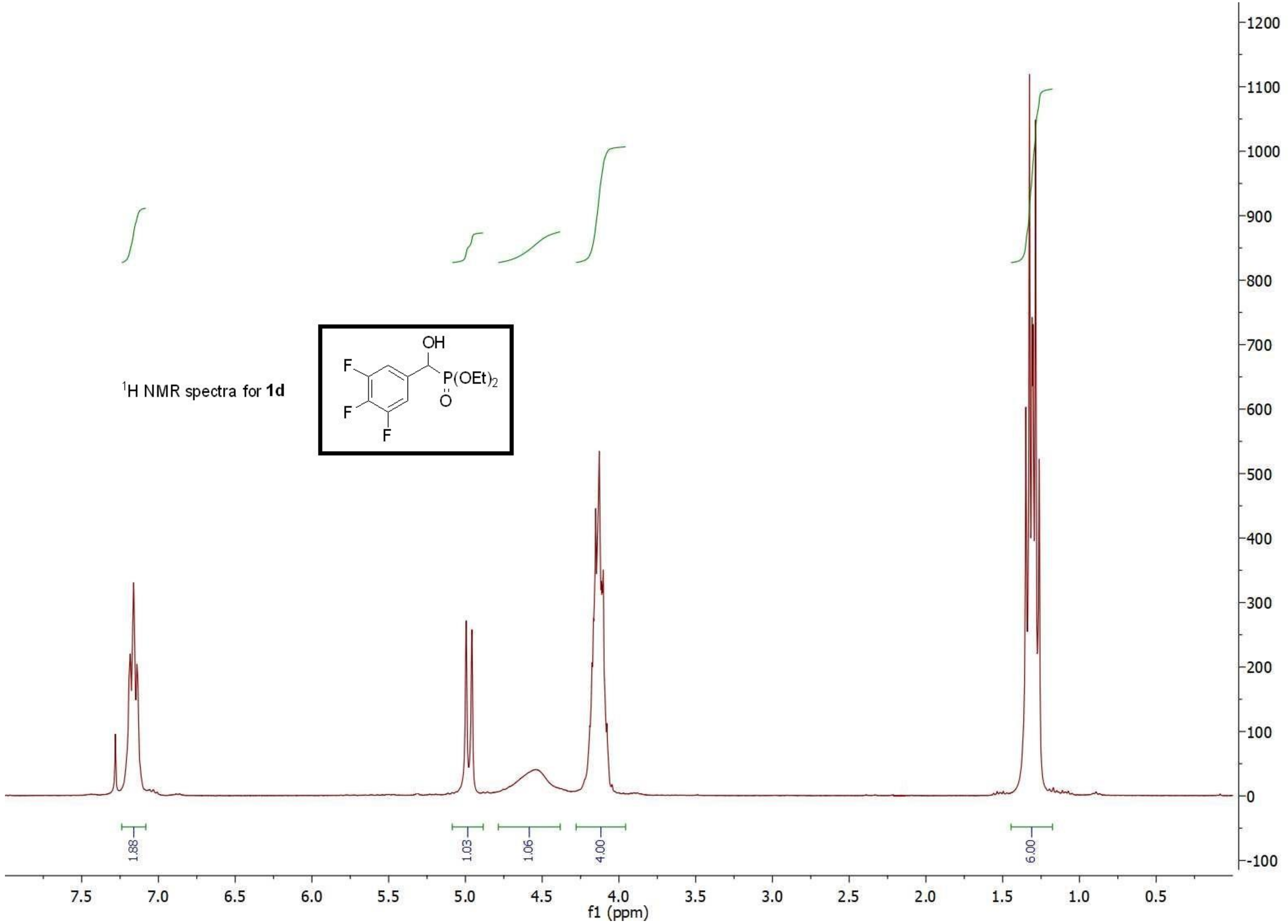

^{1}H NMR spectra for **1d**
OH
F
P(OEt)$_2$
O
F
F
1.88
1.03
1.06
4.00
6.00
7.5
7.0
6.5
6.0
5.5
5.0
4.5
4.0
3.5
3.0
2.5
2.0
1.5
1.0
0.5
f1 (ppm)
1200
1100
1000
900
800
700
600
500
400
300
200
100
0
-100

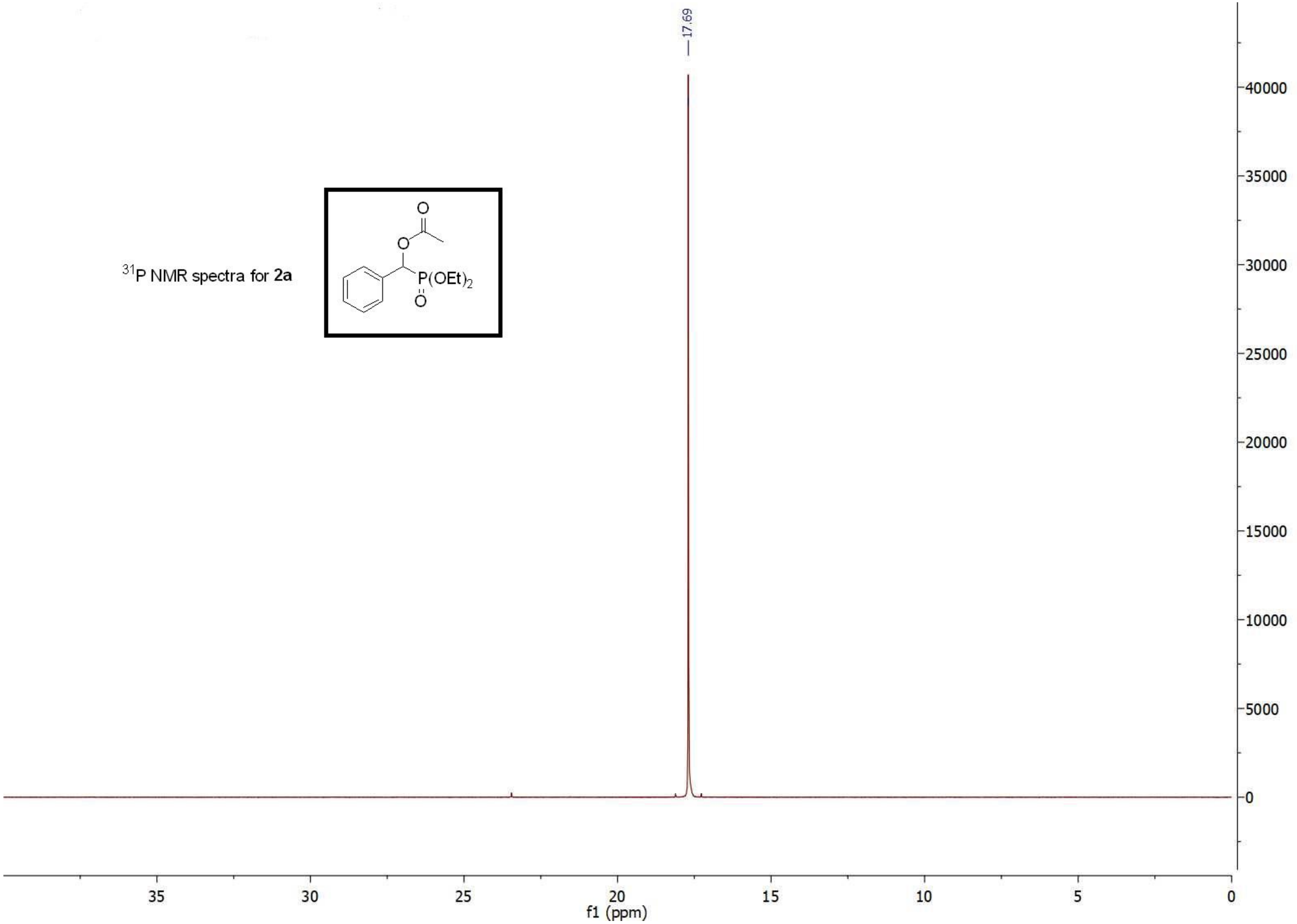

^{31}P NMR spectra for **2a**
P(OEt)$_2$
17.69
40000
35000
30000
25000
20000
15000
10000
5000
0
35
30
25
20
15
10
5
0
f1 (ppm)

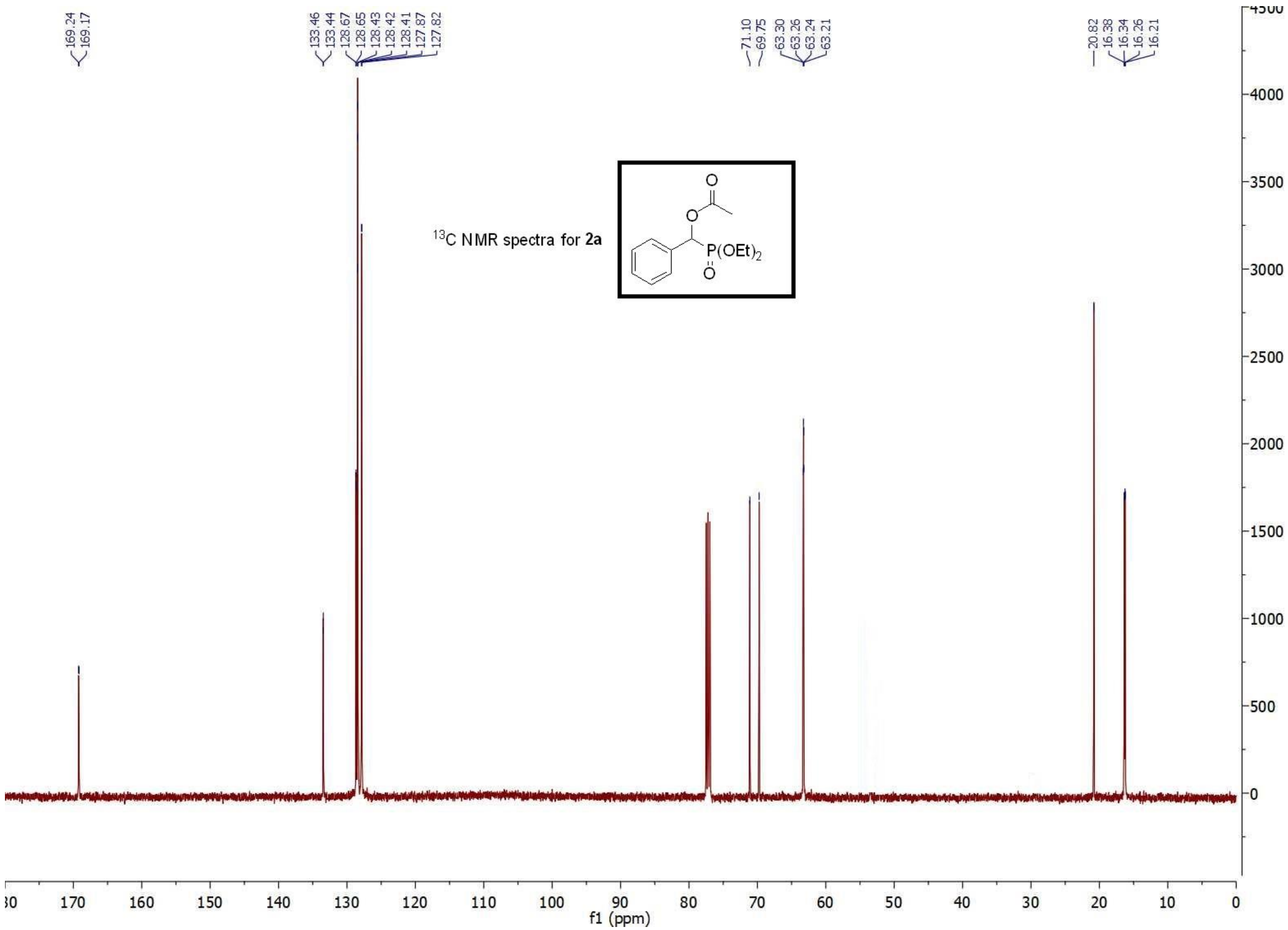

169.24
169.17
133.46
133.44
128.67
128.65
128.43
128.42
128.41
127.87
127.82
71.10
69.75
63.30
63.26
63.24
63.21
20.82
16.38
16.34
16.26
16.21
^{13}C NMR spectra for **2a**
O
O
P(OEt)$_2$
O
4500
4000
3500
3000
2500
2000
1500
1000
500
0
180
170
160
150
140
130
120
110
100
90
80
70
60
50
40
30
20
10
0
f1 (ppm)

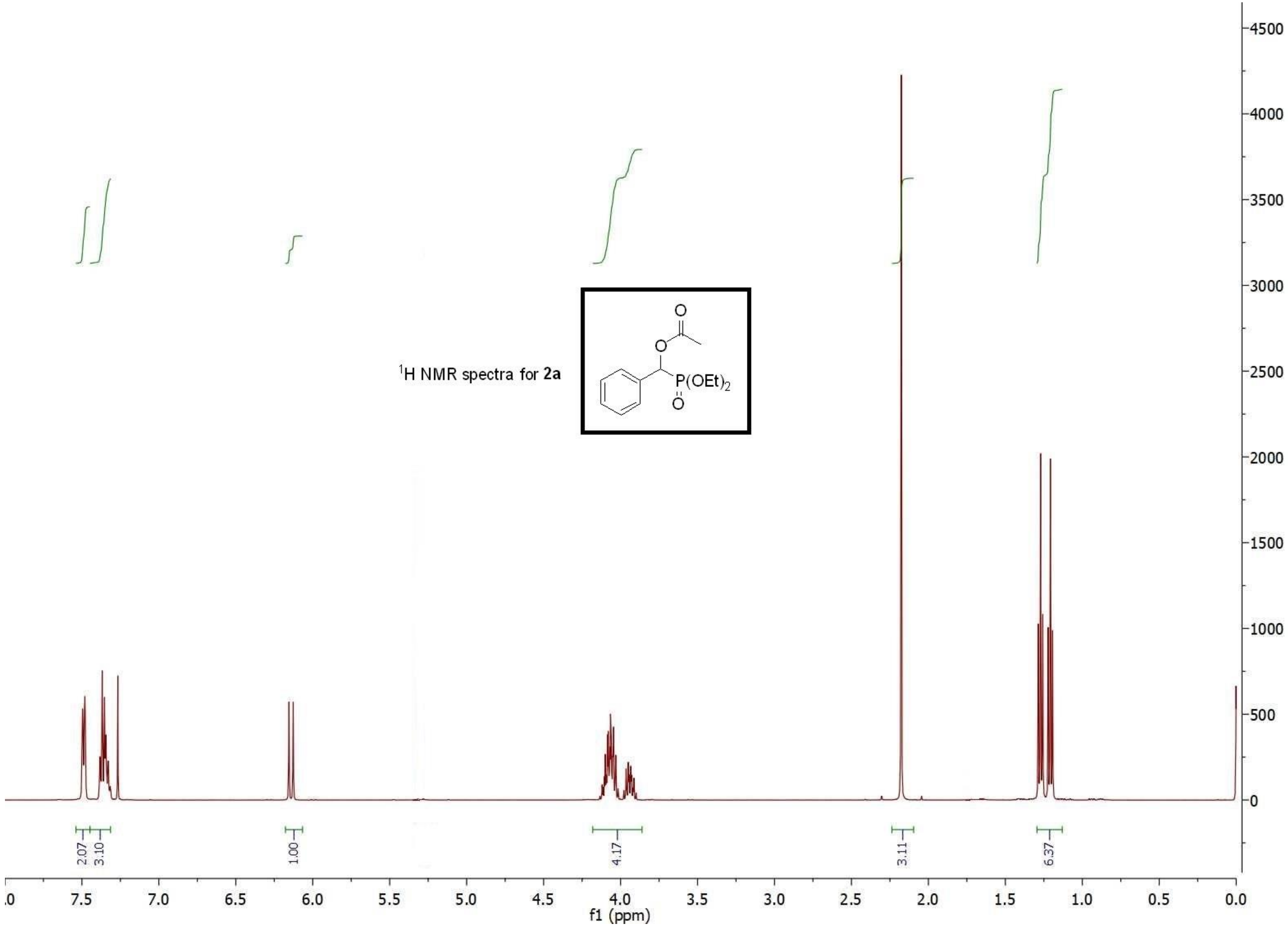

¹H NMR spectra for 2a
P(OEt)2
2.07
3.10
1.00
4.17
3.11
6.37
f1 (ppm)

^{31}P NMR spectra for **2b**

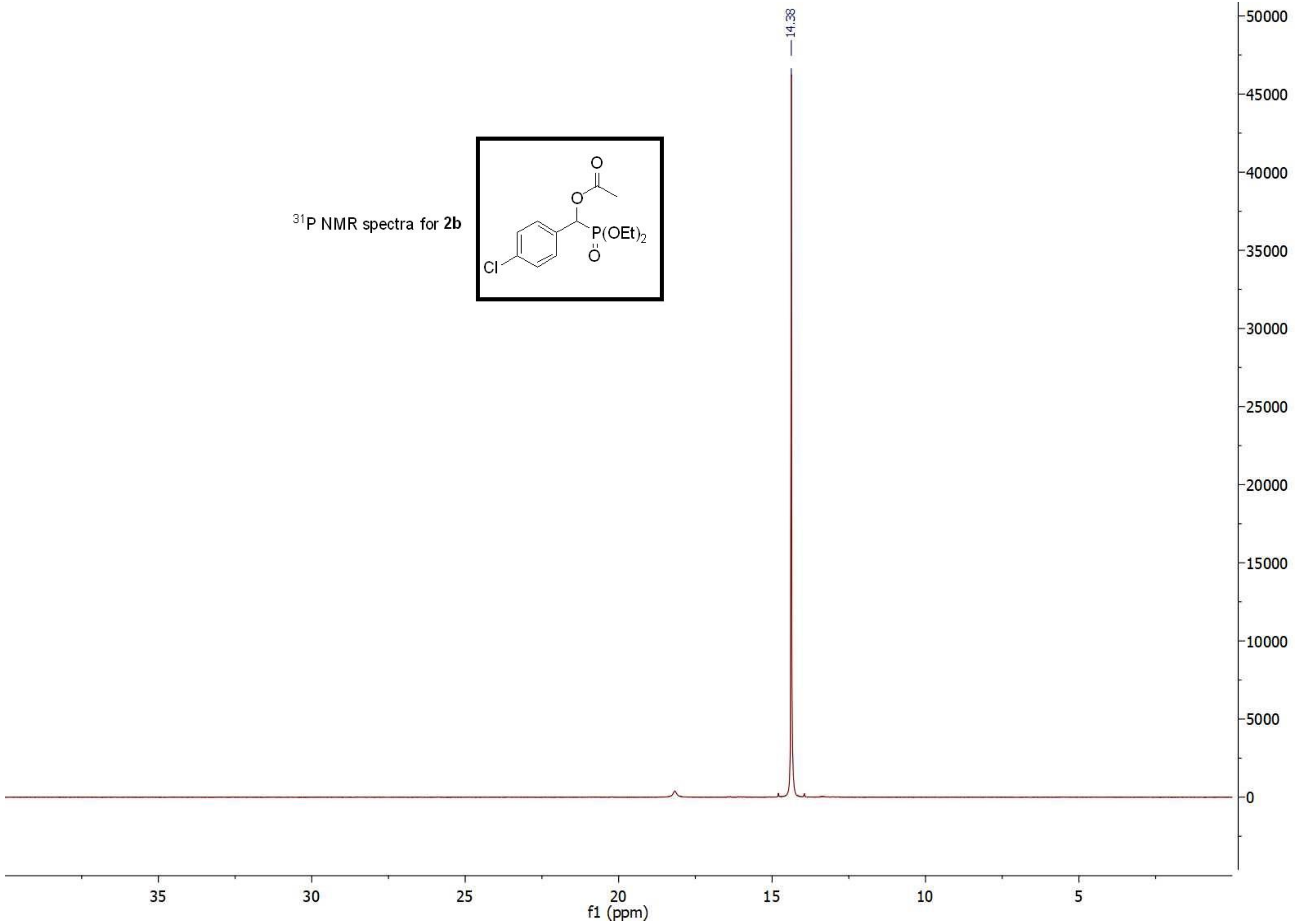

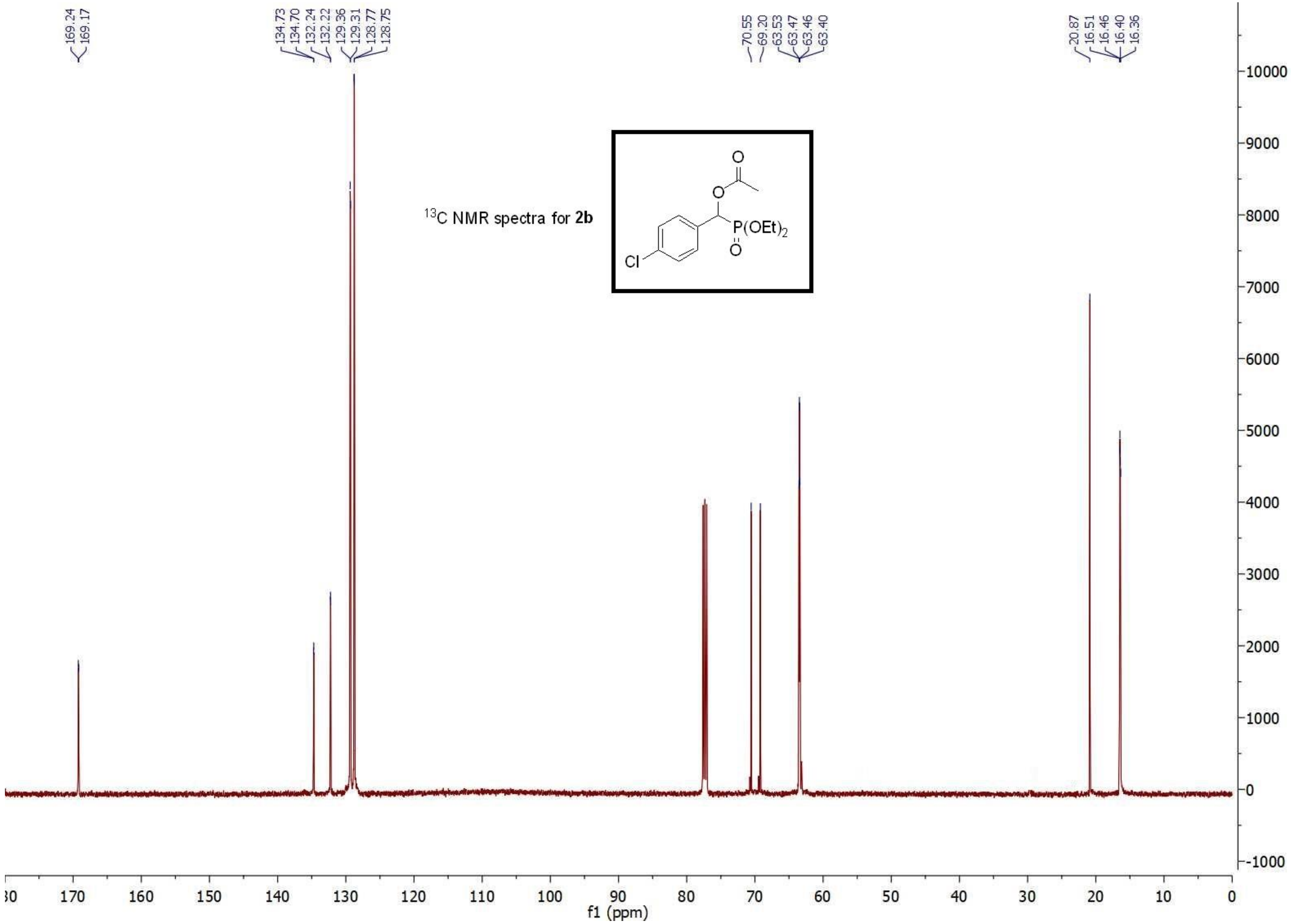
169.24
169.17
134.73
134.70
132.24
132.22
129.36
129.31
128.77
128.75
70.55
69.20
63.53
63.47
63.46
63.40
20.87
16.51
16.46
16.40
16.36
13C NMR spectra for 2b
O
O
P(OEt)2
O
Cl
10000
9000
8000
7000
6000
5000
4000
3000
2000
1000
0
-1000
170
160
150
140
130
120
110
100
90
80
70
60
50
40
30
20
10
0
f1 (ppm)

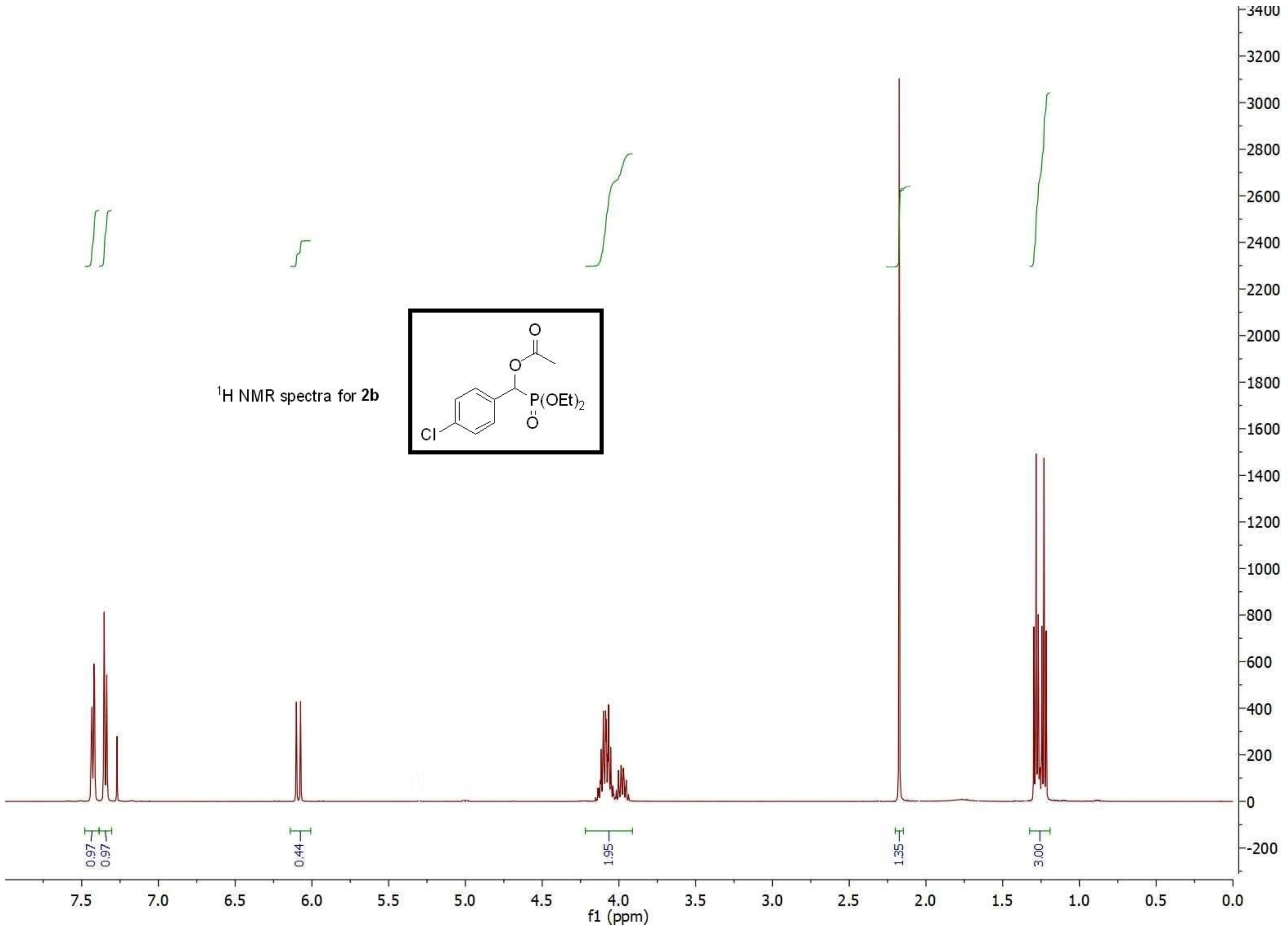

^{1}H NMR spectra for **2b**
P(OEt)$_2$
Cl
0.97
0.97
0.44
1.95
1.35
3.00
f1 (ppm)

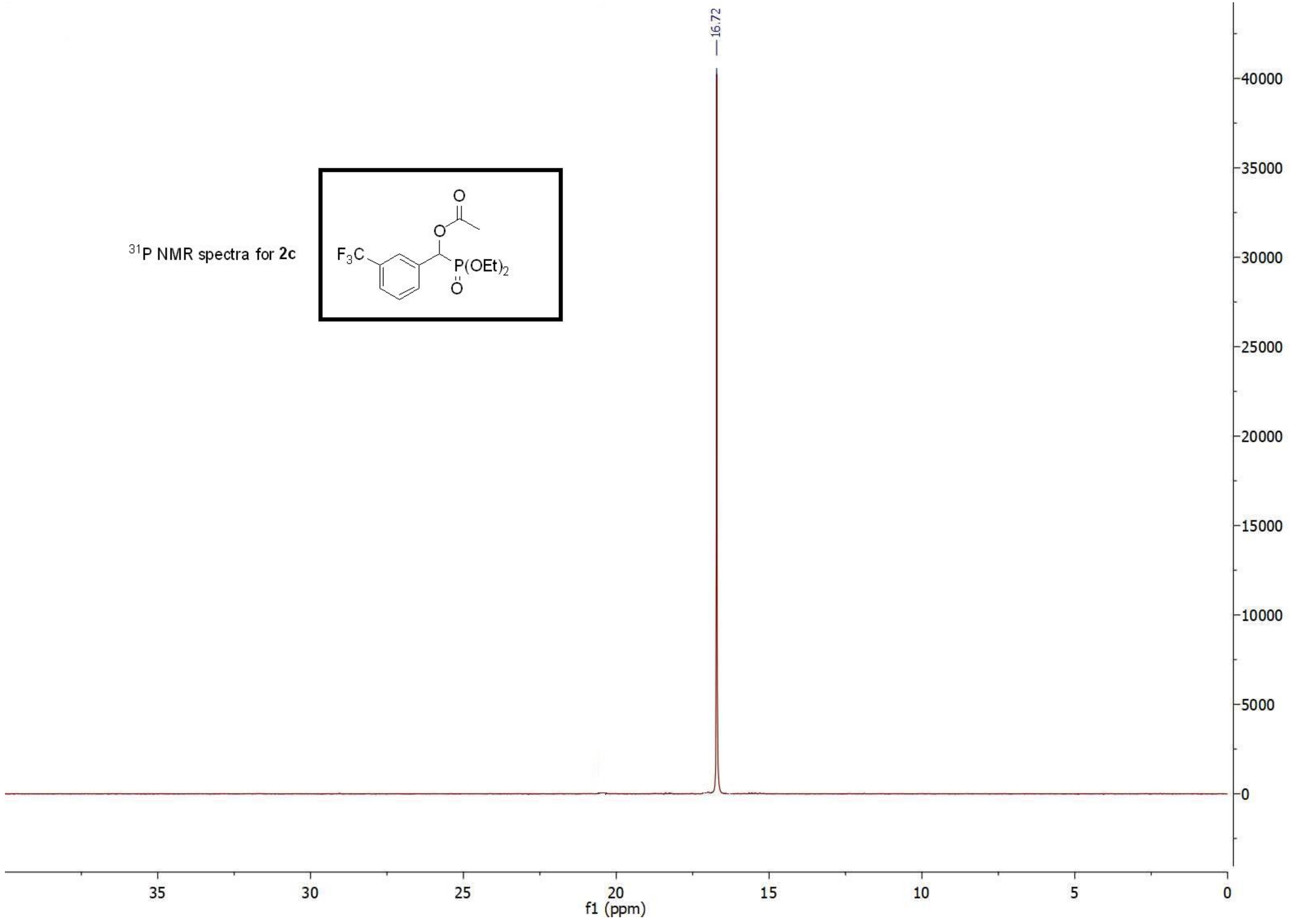

16.72
31P NMR spectra for 2c
F3C
O
O
P(OEt)2
O
40000
35000
30000
25000
20000
15000
10000
5000
0
35
30
25
20
15
10
5
0
f1 (ppm)

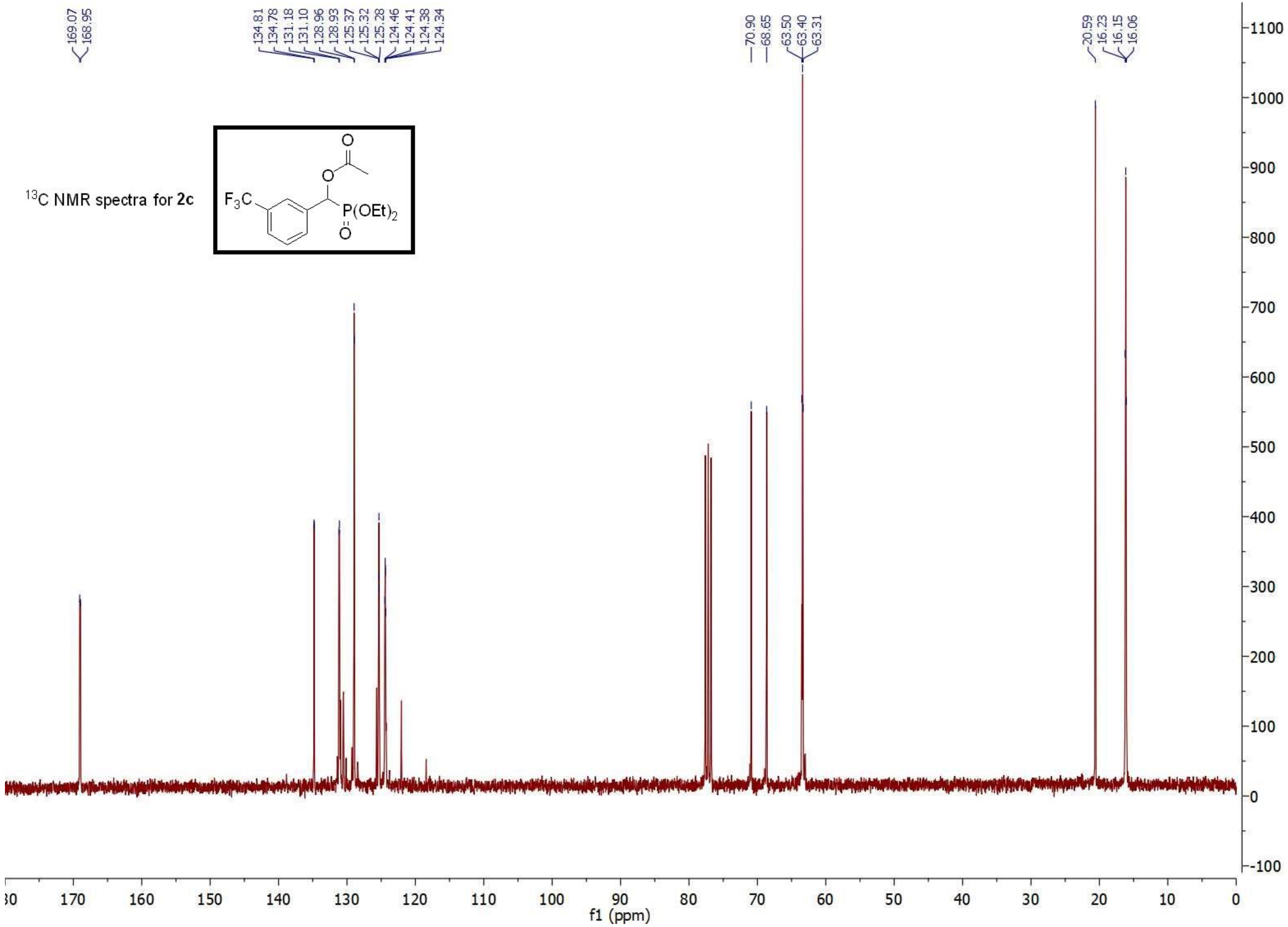

169.07
168.95
134.81
134.78
131.18
131.10
128.96
128.93
125.37
125.32
125.28
124.46
124.41
124.38
124.34
70.90
68.65
63.50
63.40
63.31
20.59
16.23
16.15
16.06
^{13}C NMR spectra for **2c**
F_3C
$P(OEt)_2$
f1 (ppm)

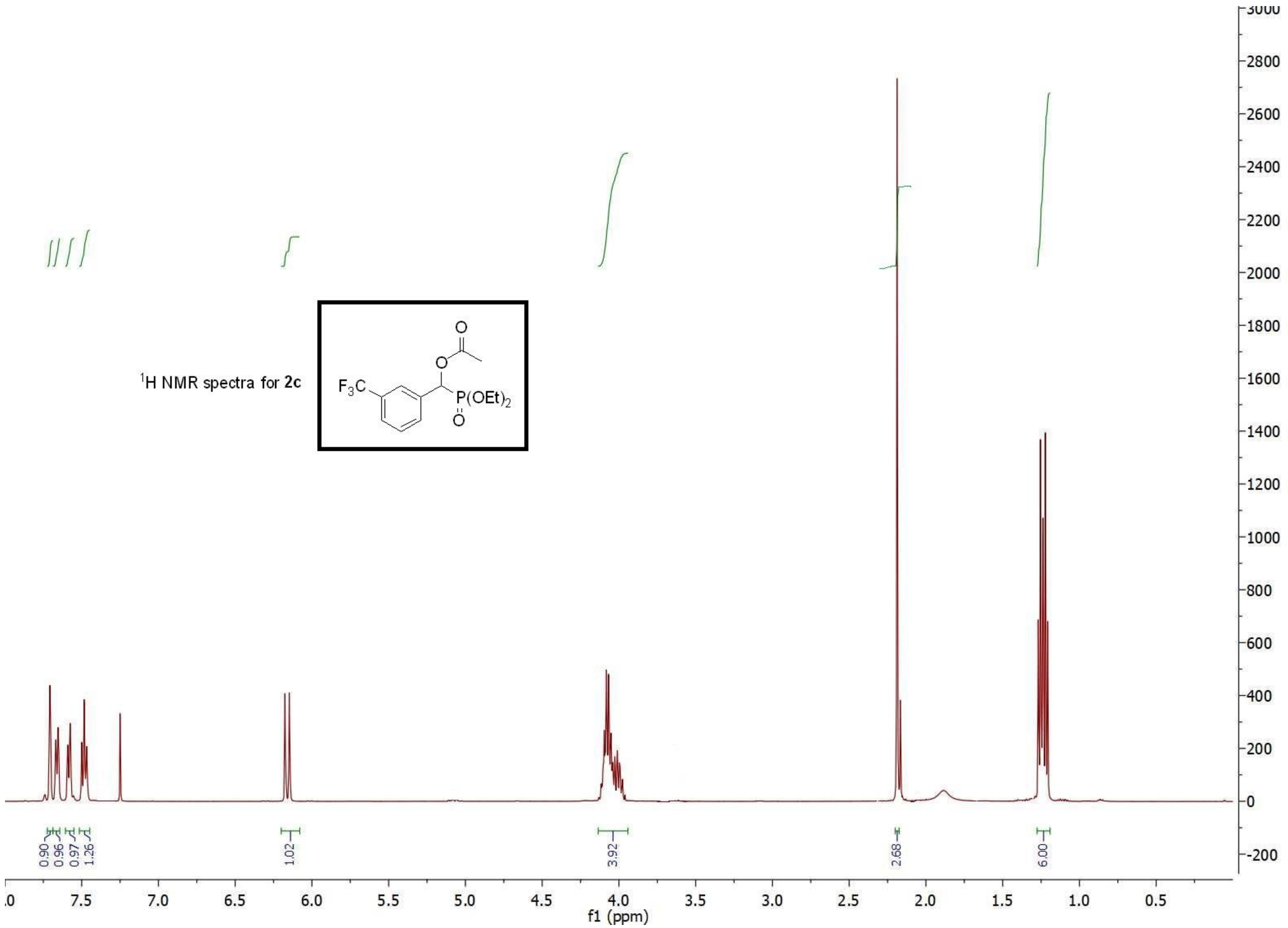

^{1}H NMR spectra for 2c
F3C
O
O
P(OEt)2
O
0.90
0.96
0.97
1.26
1.02
3.92
2.68
6.00
f1 (ppm)

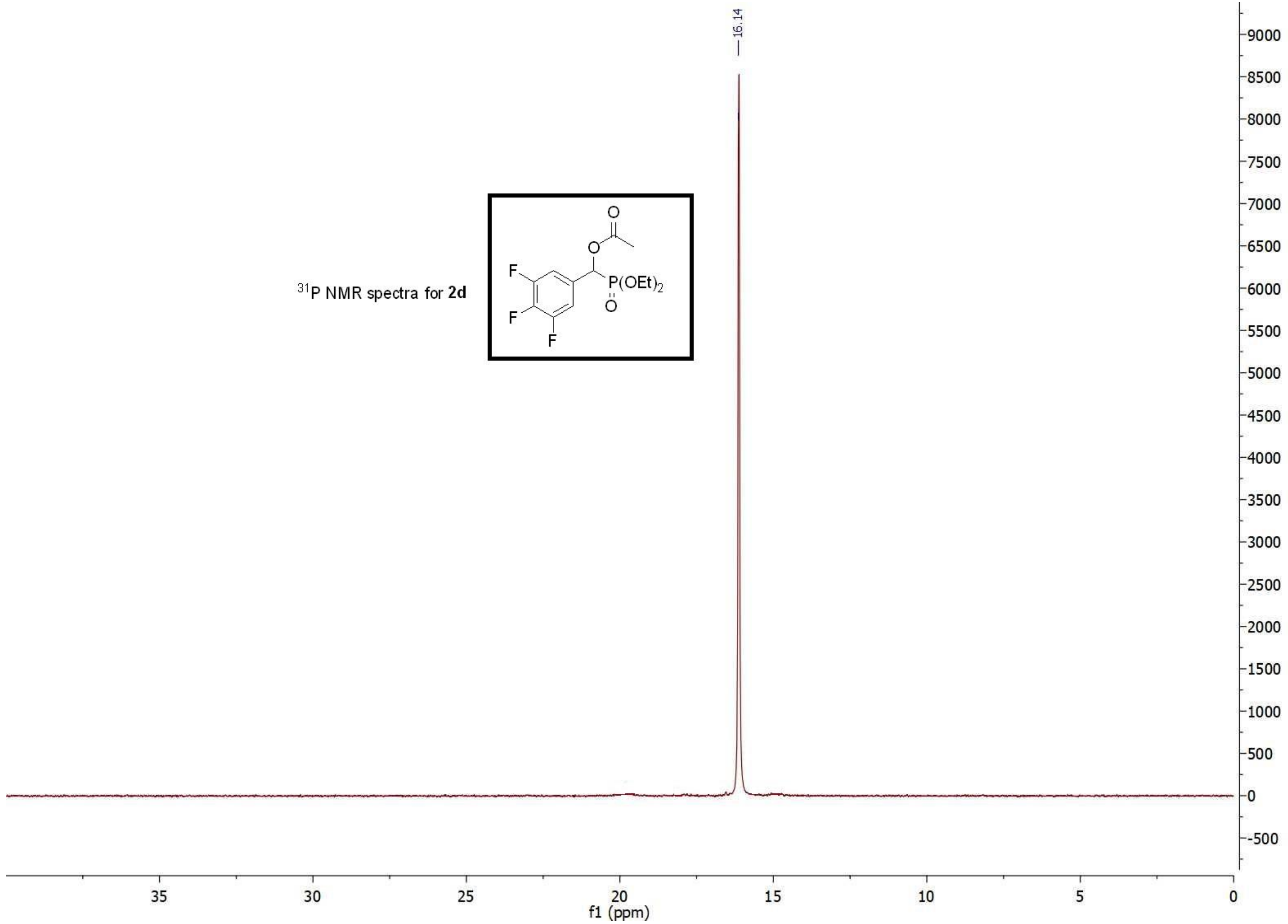

16.14
^{31}P NMR spectra for **2d**
f1 (ppm)

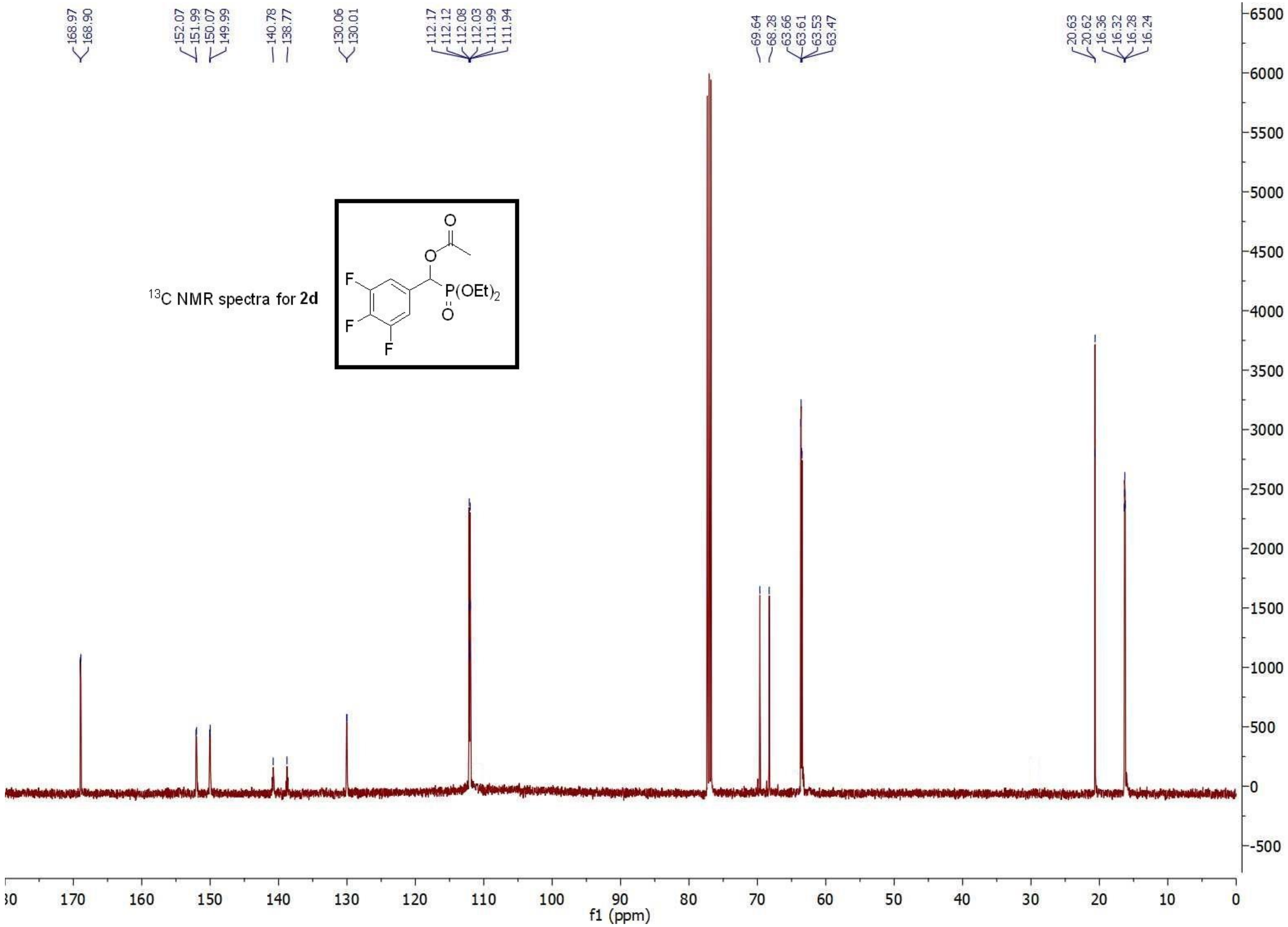

168.97
168.90
152.07
151.99
150.07
149.99
140.78
138.77
130.06
130.01
112.17
112.12
112.08
112.03
111.99
111.94
69.64
68.28
63.66
63.61
63.53
63.47
20.63
20.62
16.36
16.32
16.28
16.24
^{13}C NMR spectra for **2d**
F
F
F
O
O
P(OEt)$_2$
O
O
f1 (ppm)

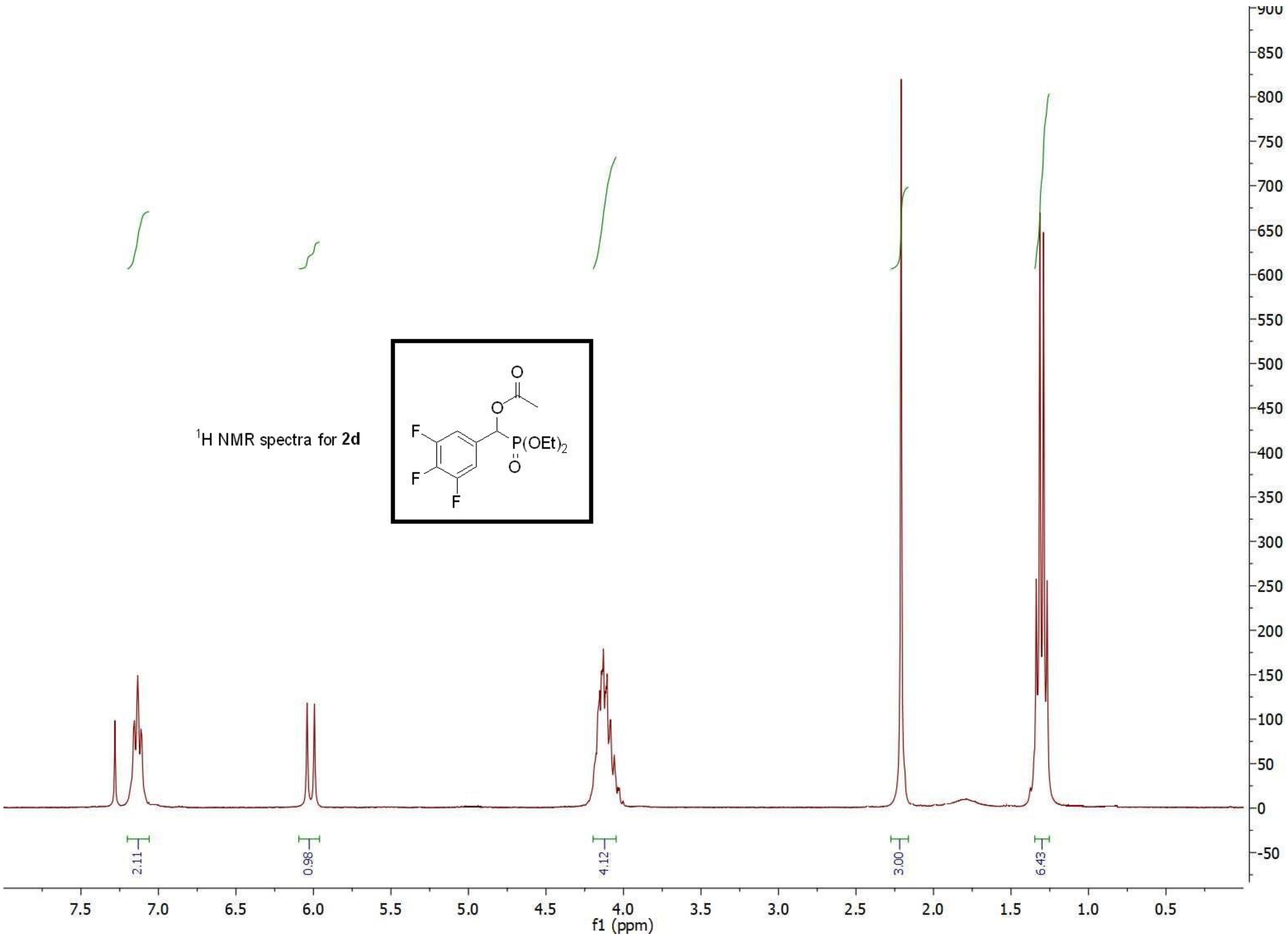

^{1}H NMR spectra for **2d**
F
F
F
O
O
P(OEt)2
O
2.11
0.98
4.12
3.00
6.43
f1 (ppm)

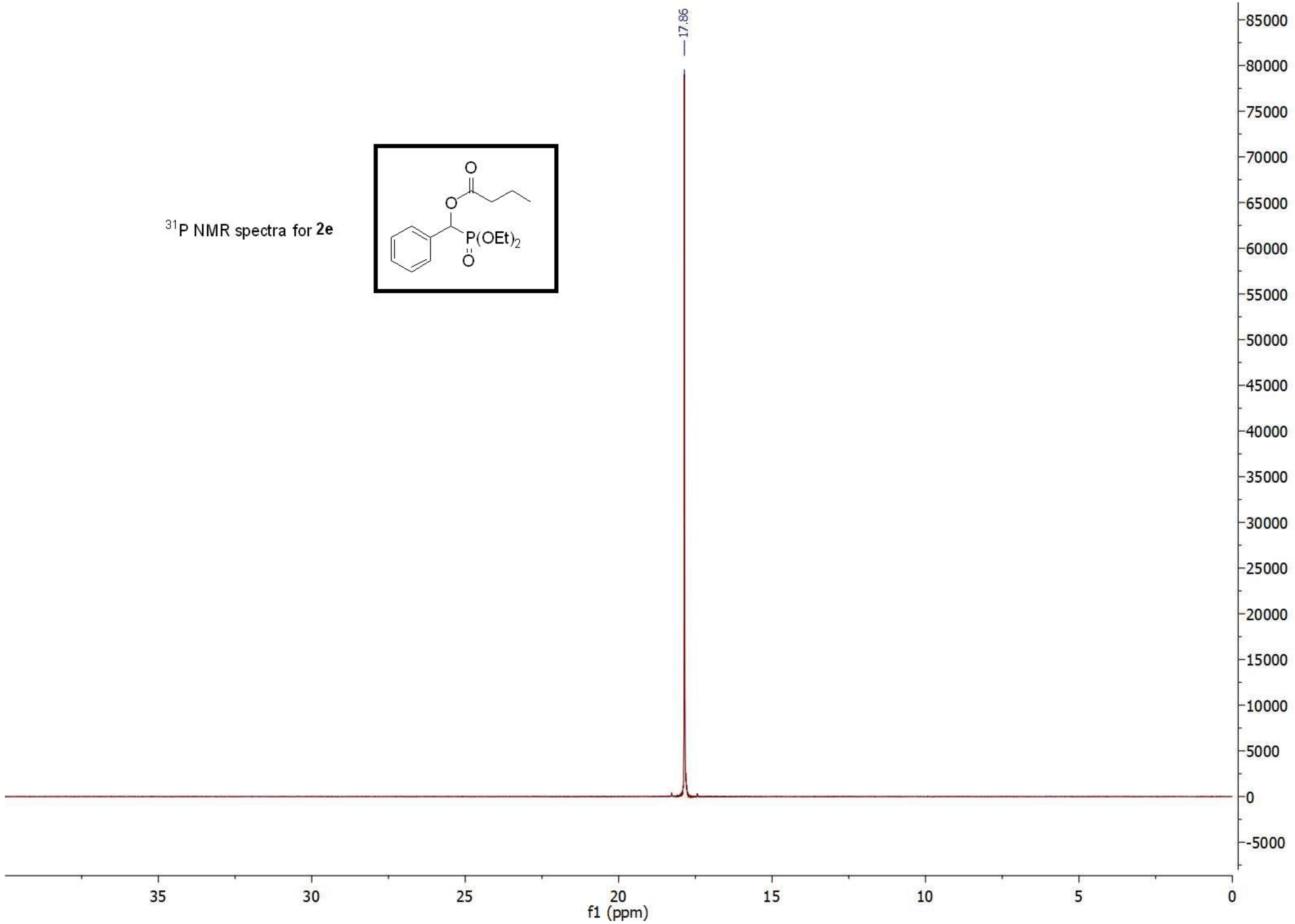

17.86
^{31}P NMR spectra for **2e**
O
O
P(OEt)2
O
85000
80000
75000
70000
65000
60000
55000
50000
45000
40000
35000
30000
25000
20000
15000
10000
5000
0
-5000
35
30
25
20
15
10
5
0
f1 (ppm)

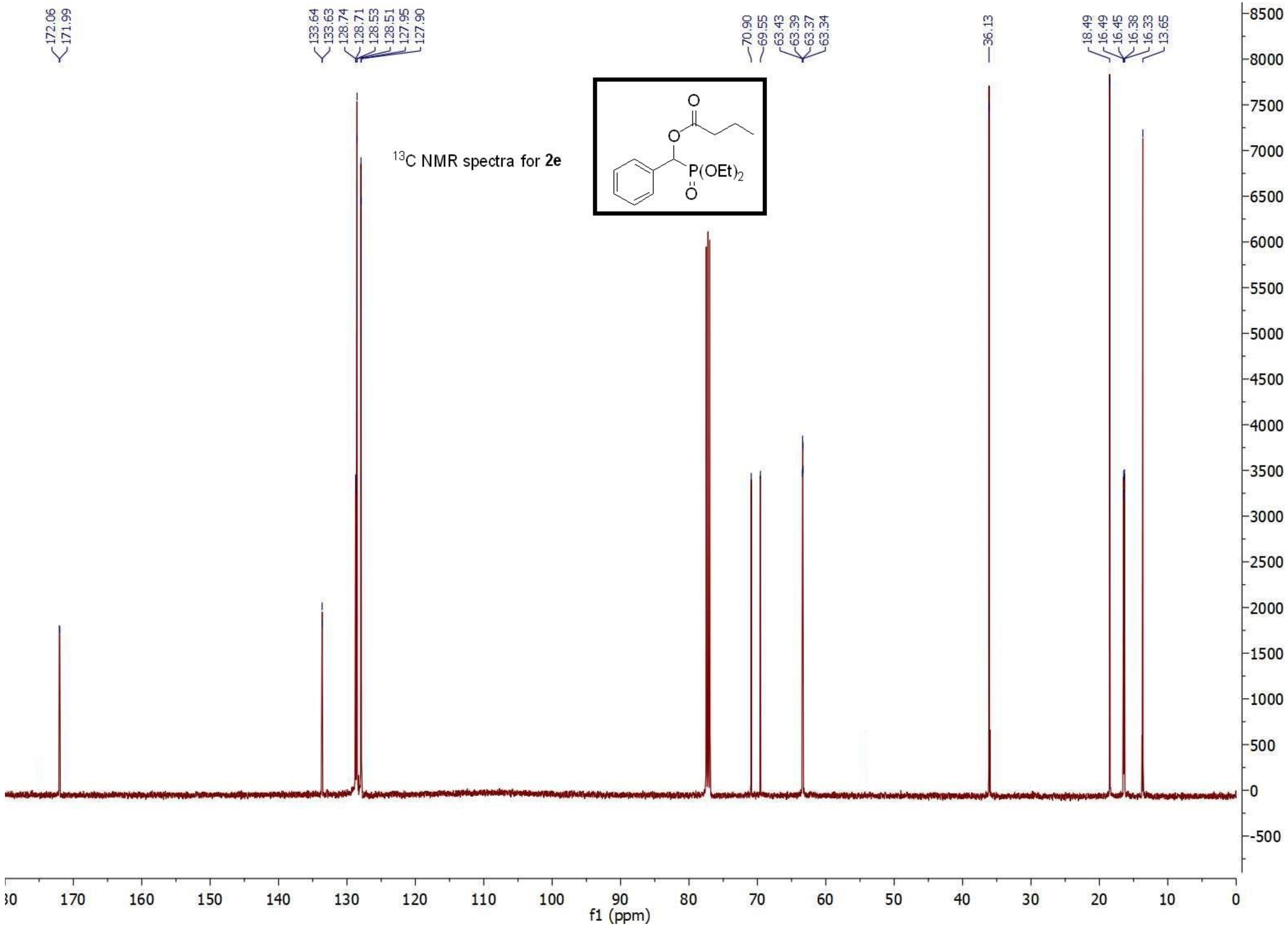
172.06
171.99
133.64
133.63
128.74
128.71
128.53
128.51
127.95
127.90
70.90
69.55
63.43
63.39
63.37
63.34
36.13
18.49
16.49
16.45
16.38
16.33
13.65
13C NMR spectra for 2e
P(OEt)2
f1 (ppm)

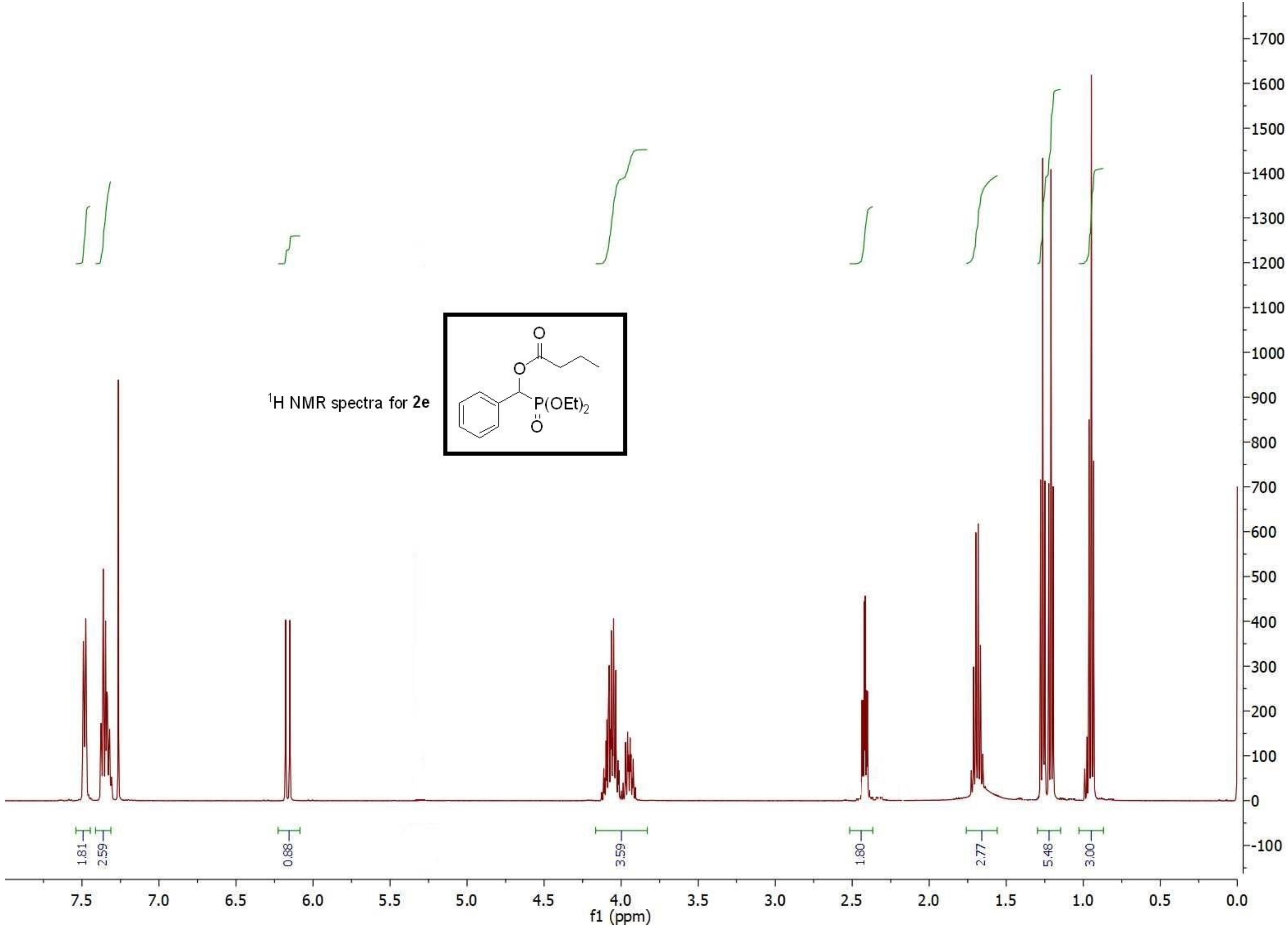

^{1}H NMR spectra for **2e**
O
O
P(OEt)2
O
1.81
2.59
0.88
3.59
1.80
2.77
5.48
3.00
7.5
7.0
6.5
6.0
5.5
5.0
4.5
4.0
3.5
3.0
2.5
2.0
1.5
1.0
0.5
0.0
f1 (ppm)
1700
1600
1500
1400
1300
1200
1100
1000
900
800
700
600
500
400
300
200
100
0
-100

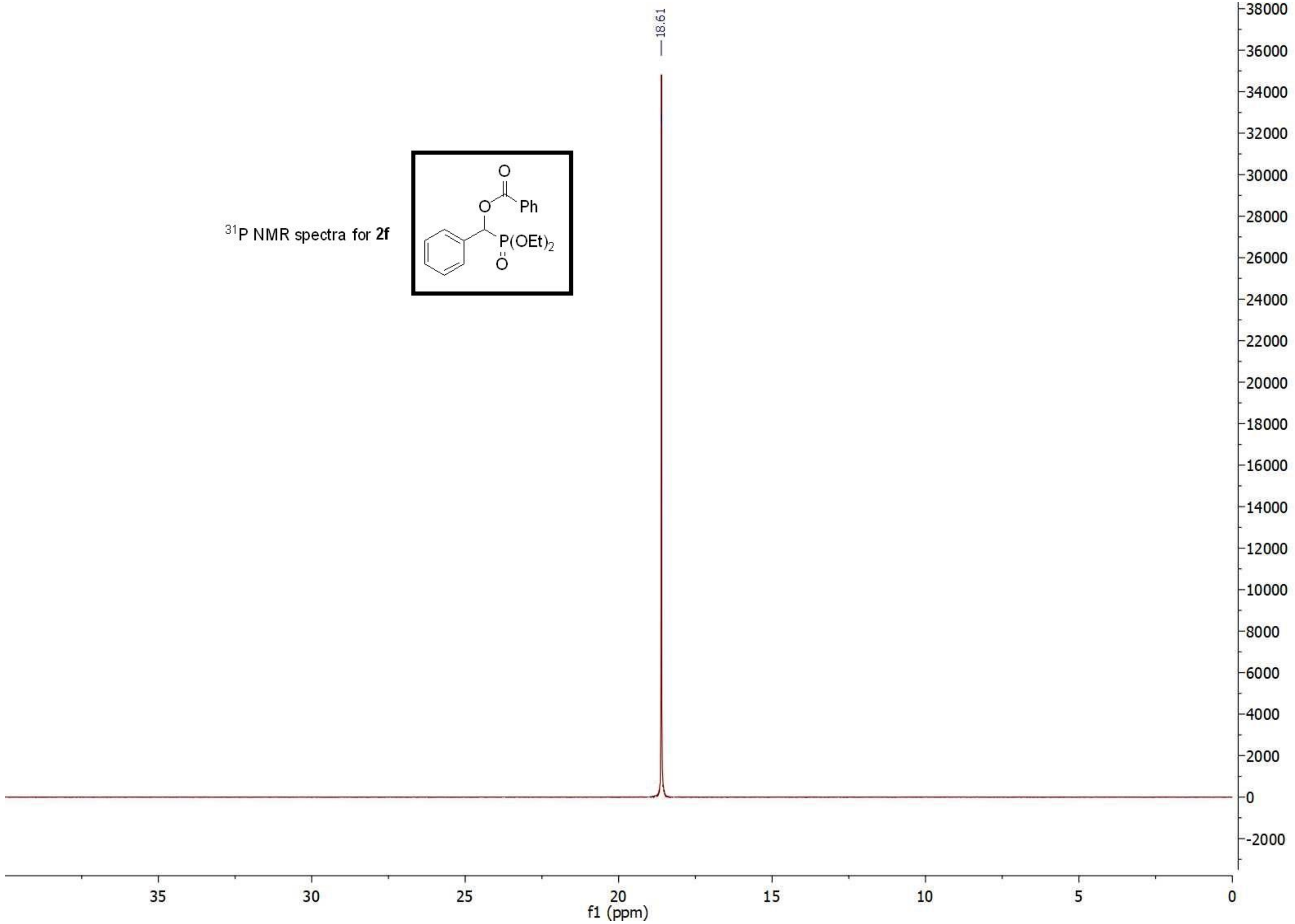

18.61
^{31}P NMR spectra for **2f**
Ph
P(OEt)$_2$
f1 (ppm)

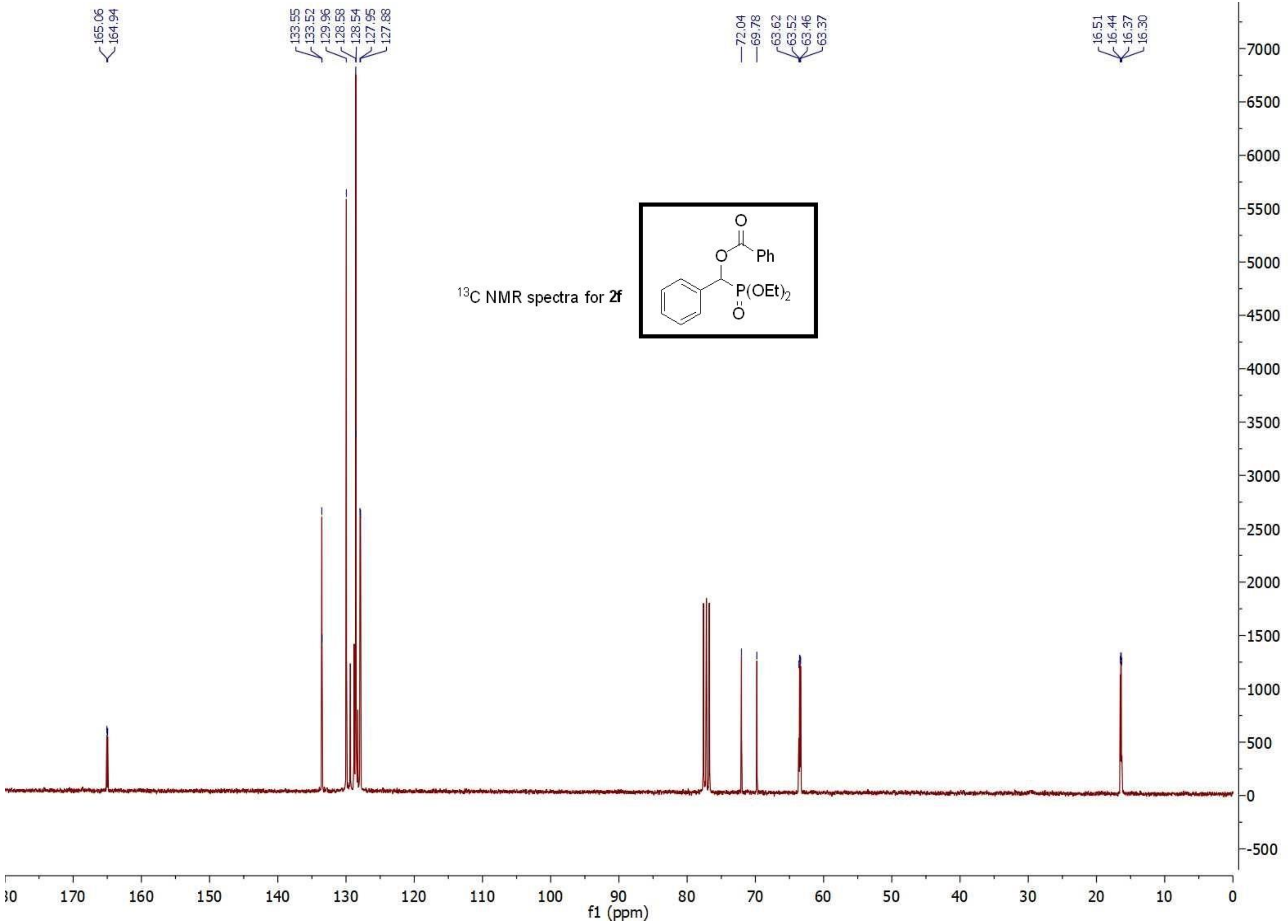

165.06
164.94
133.55
133.52
129.96
128.58
128.54
127.95
127.88
72.04
69.78
63.62
63.52
63.46
63.37
16.51
16.44
16.37
16.30
^{13}C NMR spectra for 2f
O
O
Ph
P(OEt)2
O
f1 (ppm)

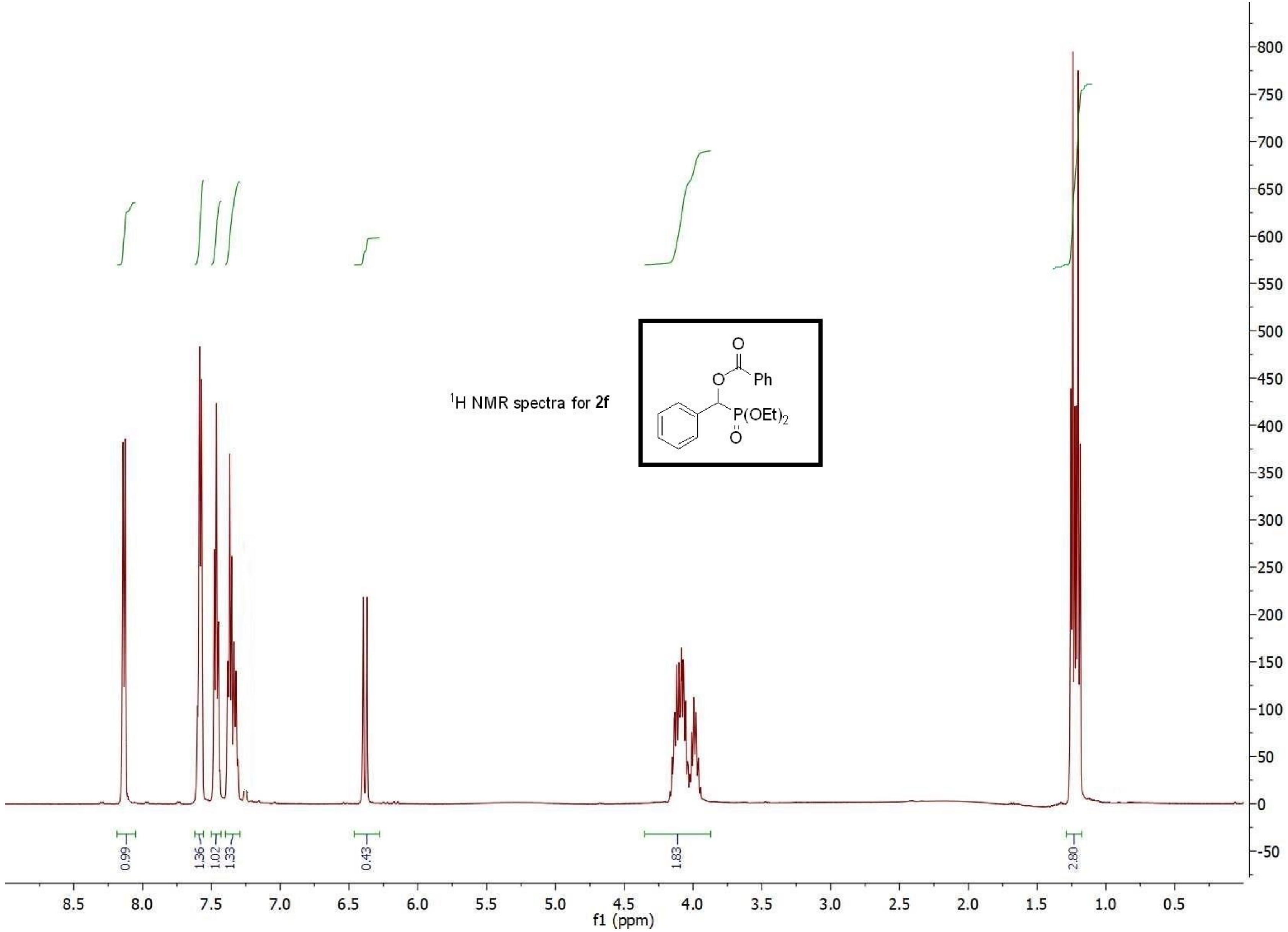

1H NMR spectra for 2f
Ph
P(OEt)2
0.99
1.36
1.02
1.33
0.43
1.83
2.80
f1 (ppm)

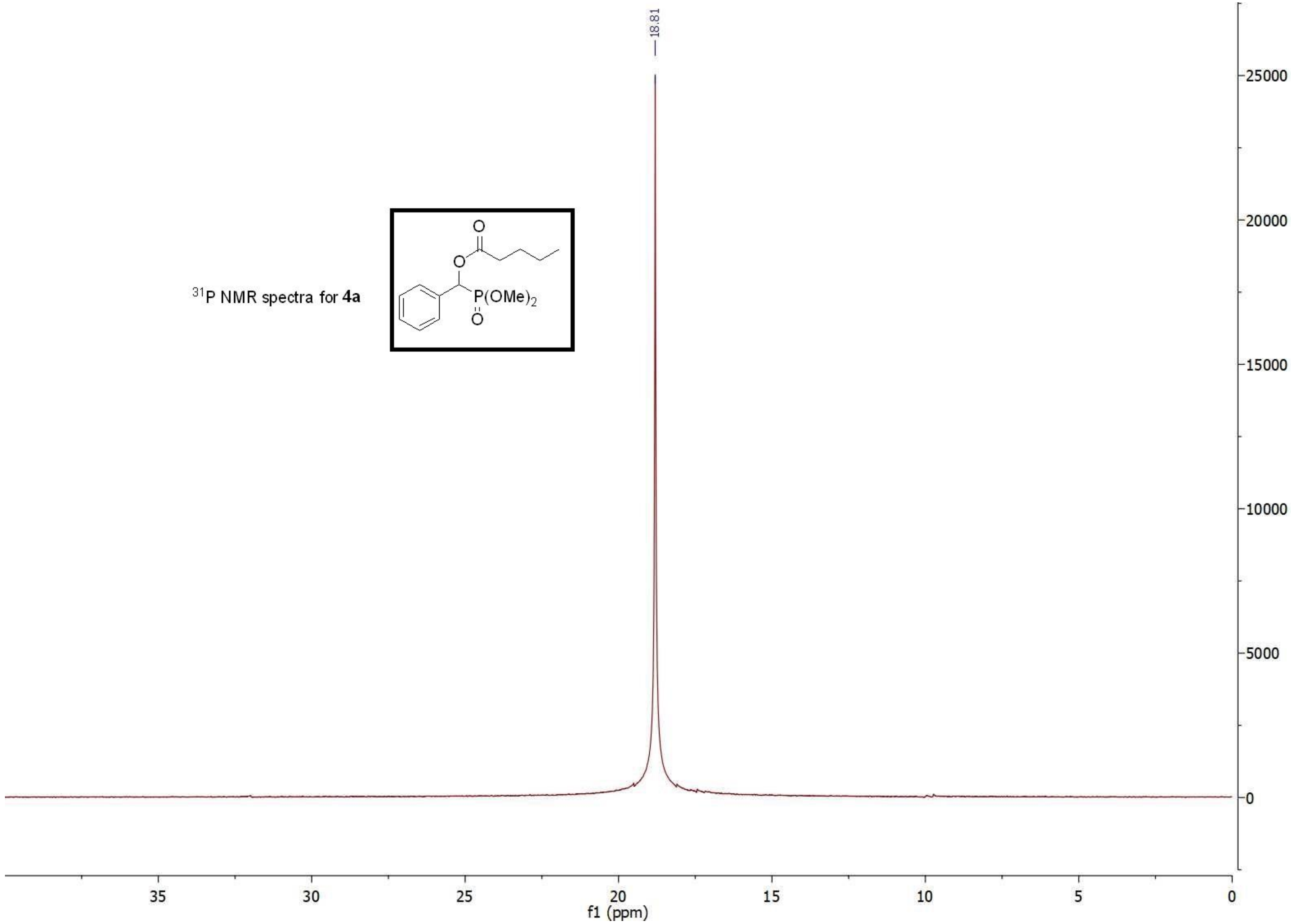
18.81
^{31}P NMR spectra for **4a**
O
O
P(OMe)$_2$
O
25000
20000
15000
10000
5000
0
35
30
25
20
15
10
5
0
f1 (ppm)

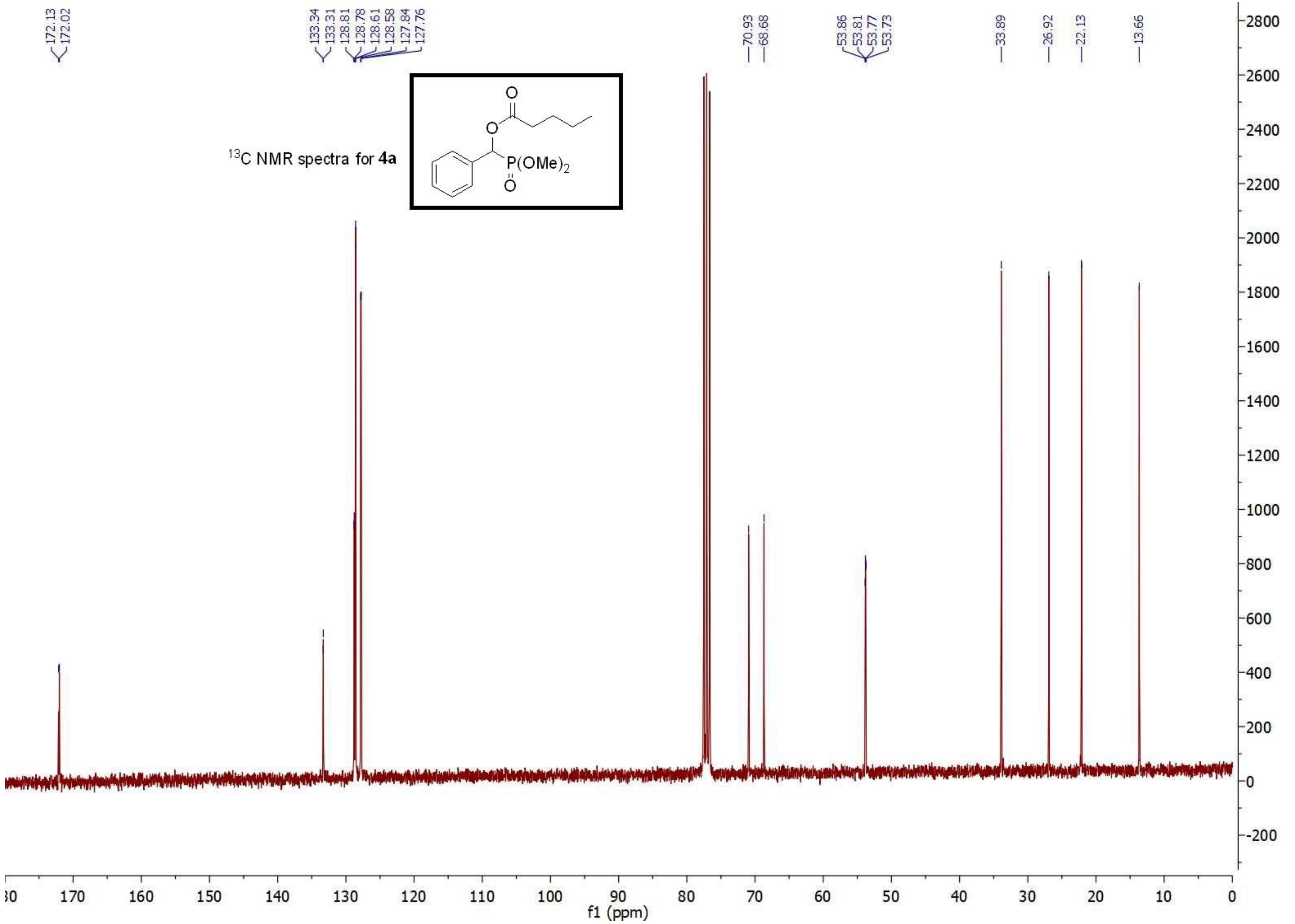

172.13
172.02
133.34
133.31
128.81
128.78
128.61
128.58
127.84
127.76
70.93
68.68
53.86
53.81
53.77
53.73
33.89
26.92
22.13
13.66
^{13}C NMR spectra for **4a**
P(OMe)$_2$
f1 (ppm)

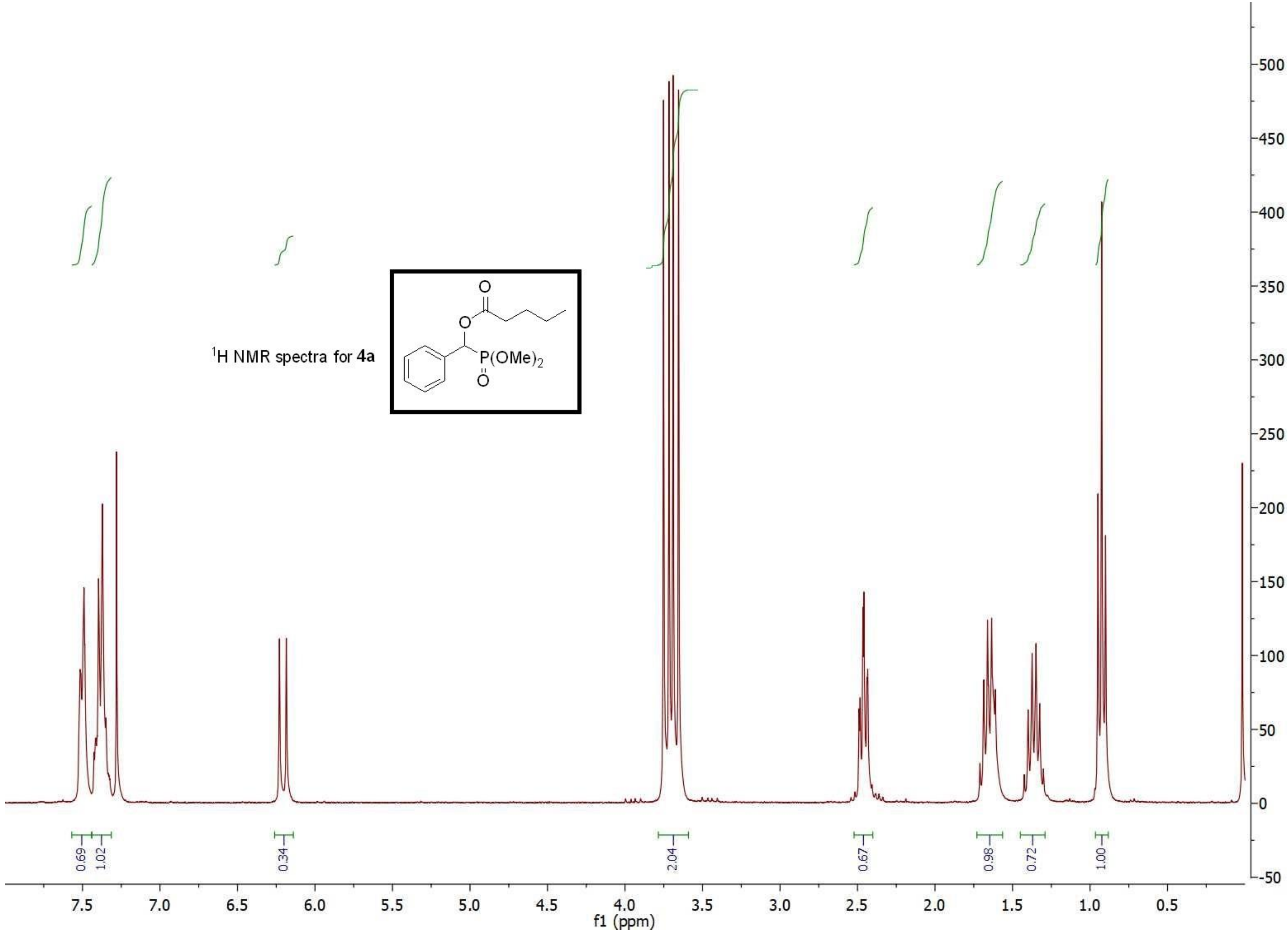

¹H NMR spectra for **4a**
P(OMe)2
f1 (ppm)
0.69
1.02
0.34
2.04
0.67
0.98
0.72
1.00

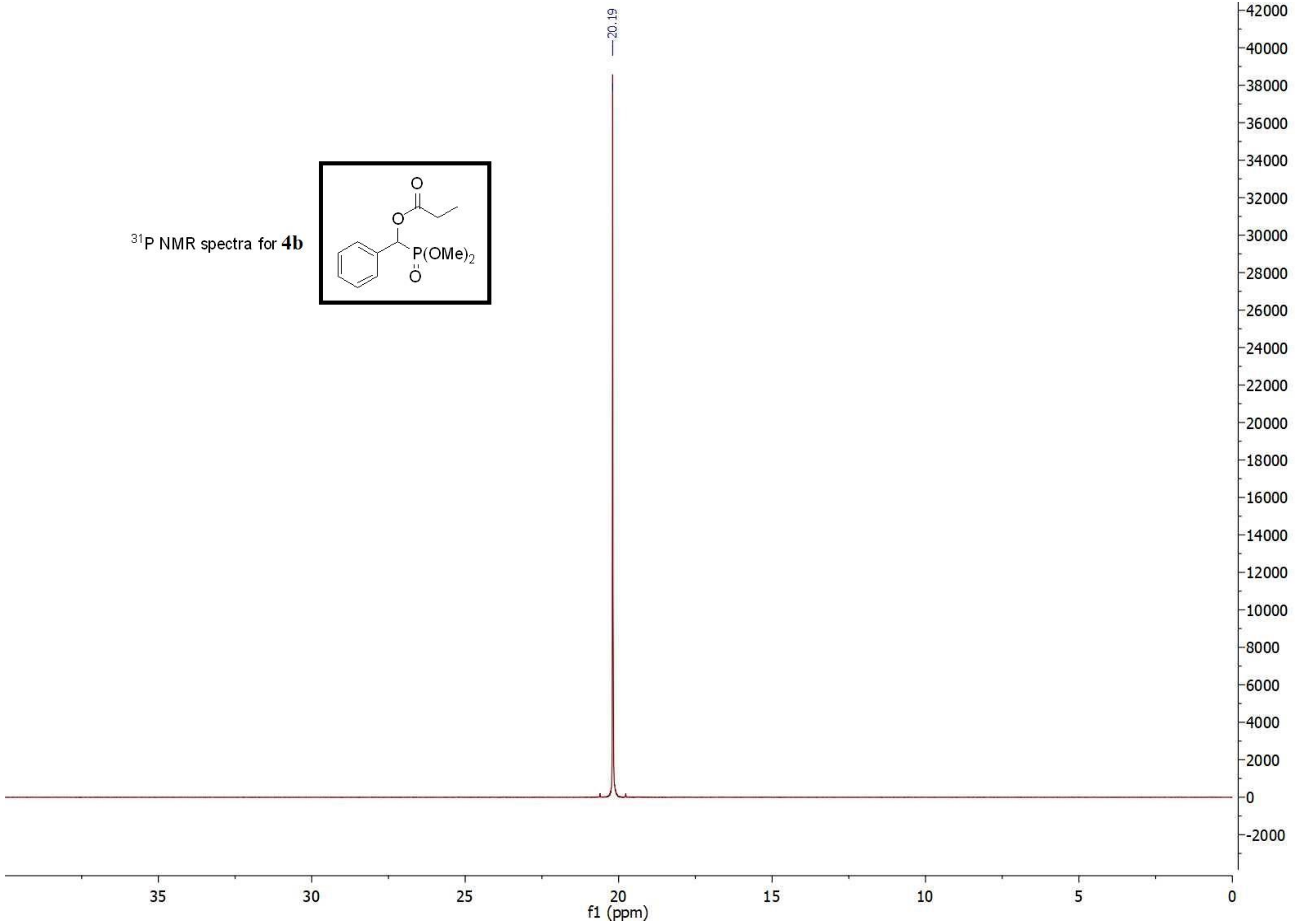

^{31}P NMR spectra for **4b**

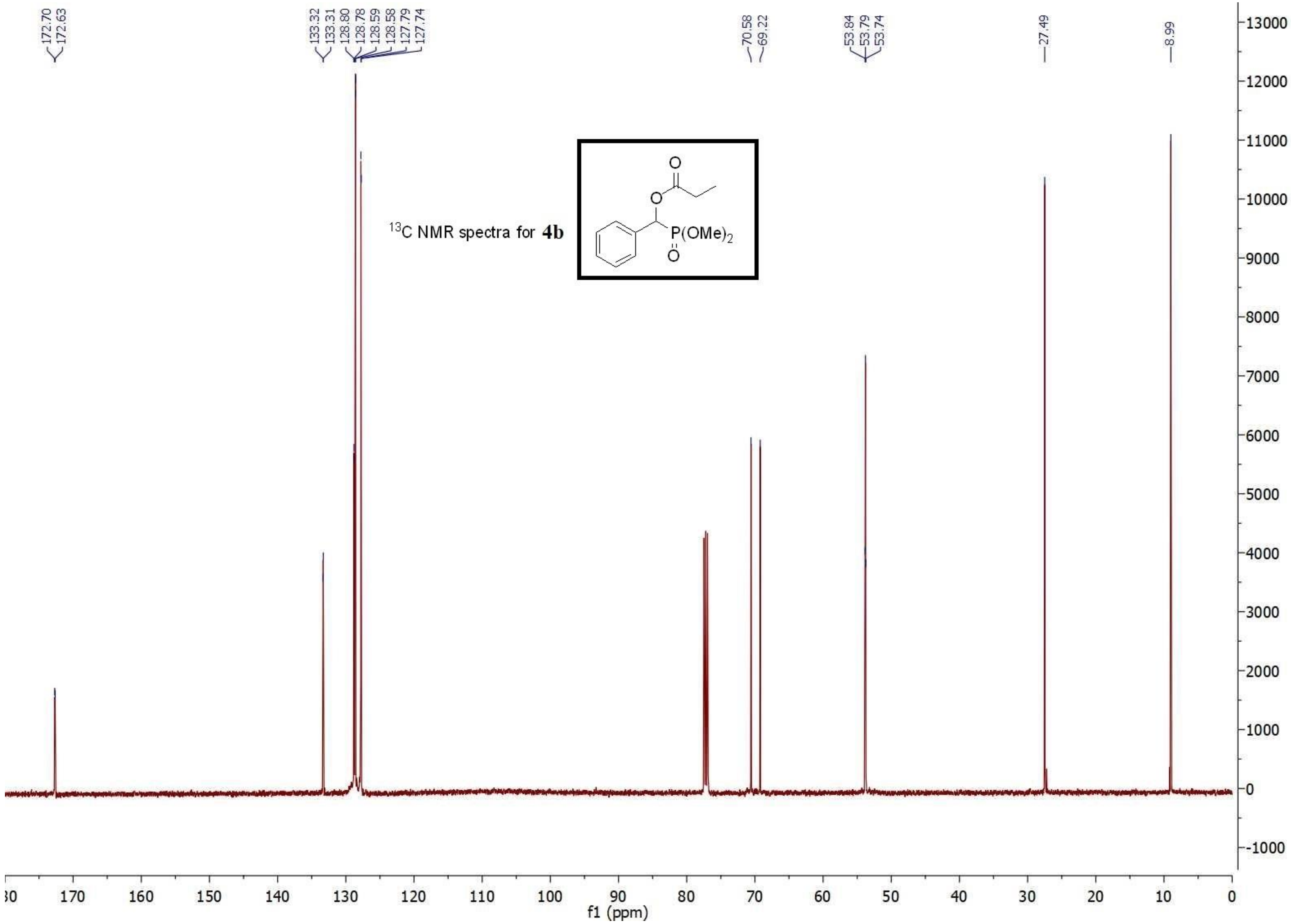
172.70
172.63
133.32
133.31
128.80
128.78
128.59
128.58
127.79
127.74
70.58
69.22
53.84
53.79
53.74
27.49
8.99
13C NMR spectra for 4b
O
O
P(OMe)2
O
f1 (ppm)

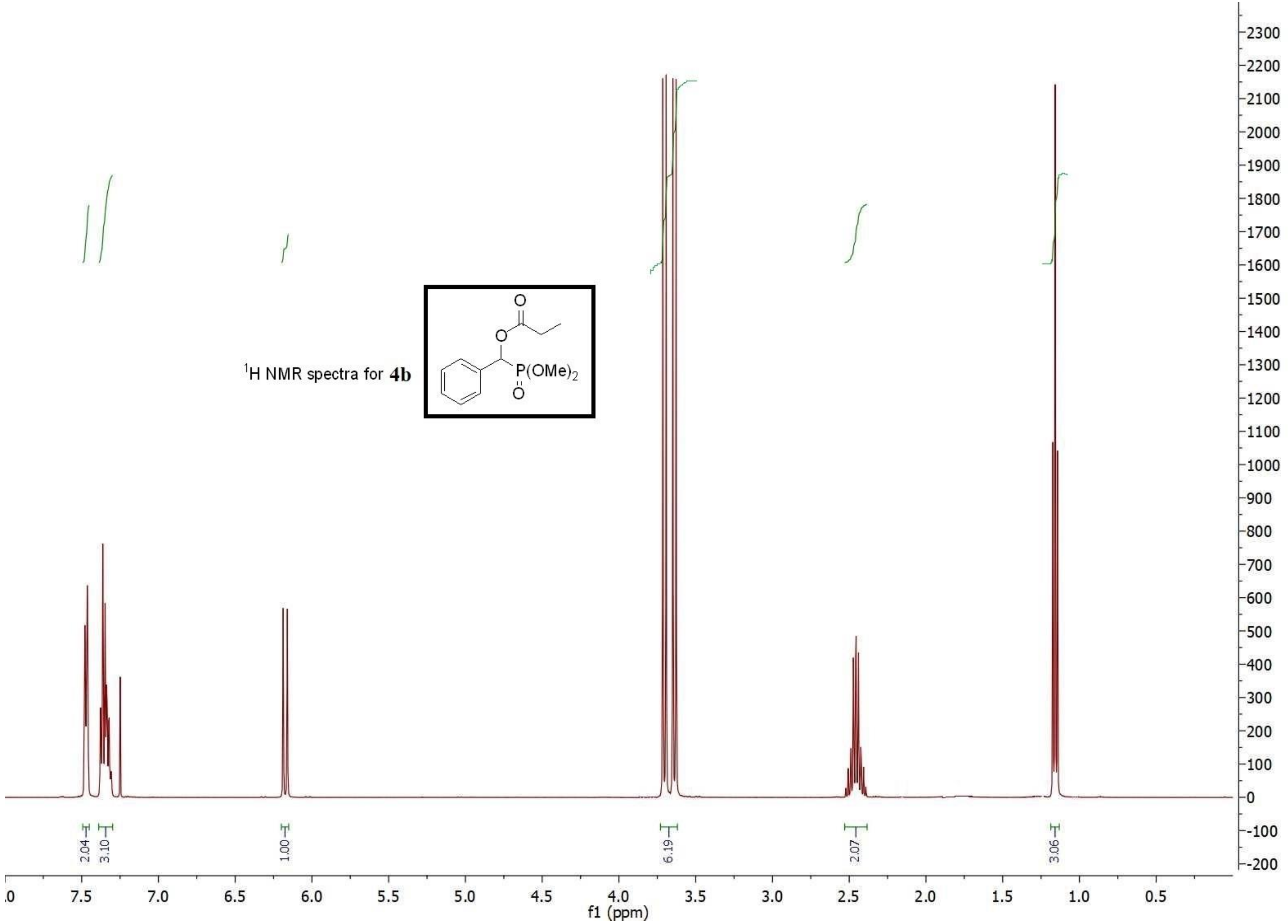

¹H NMR spectra for 4b
P(OMe)2
O
O
O
2.04
3.10
1.00
6.19
2.07
3.06
f1 (ppm)

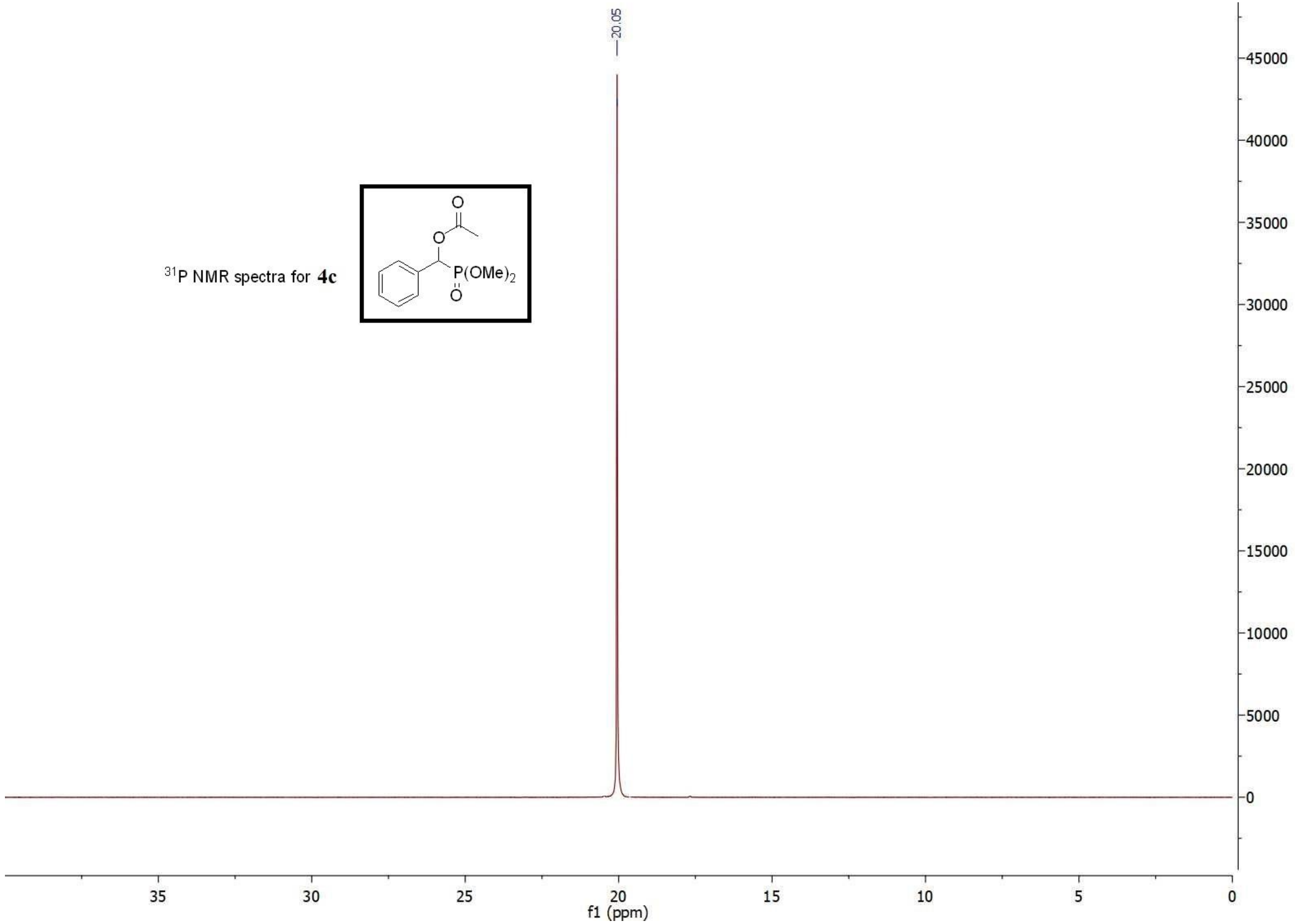

^{31}P NMR spectra for **4c**
P(OMe)$_2$
O
20.05
45000
40000
35000
30000
25000
20000
15000
10000
5000
0
35
30
25
20
15
10
5
0
f1 (ppm)

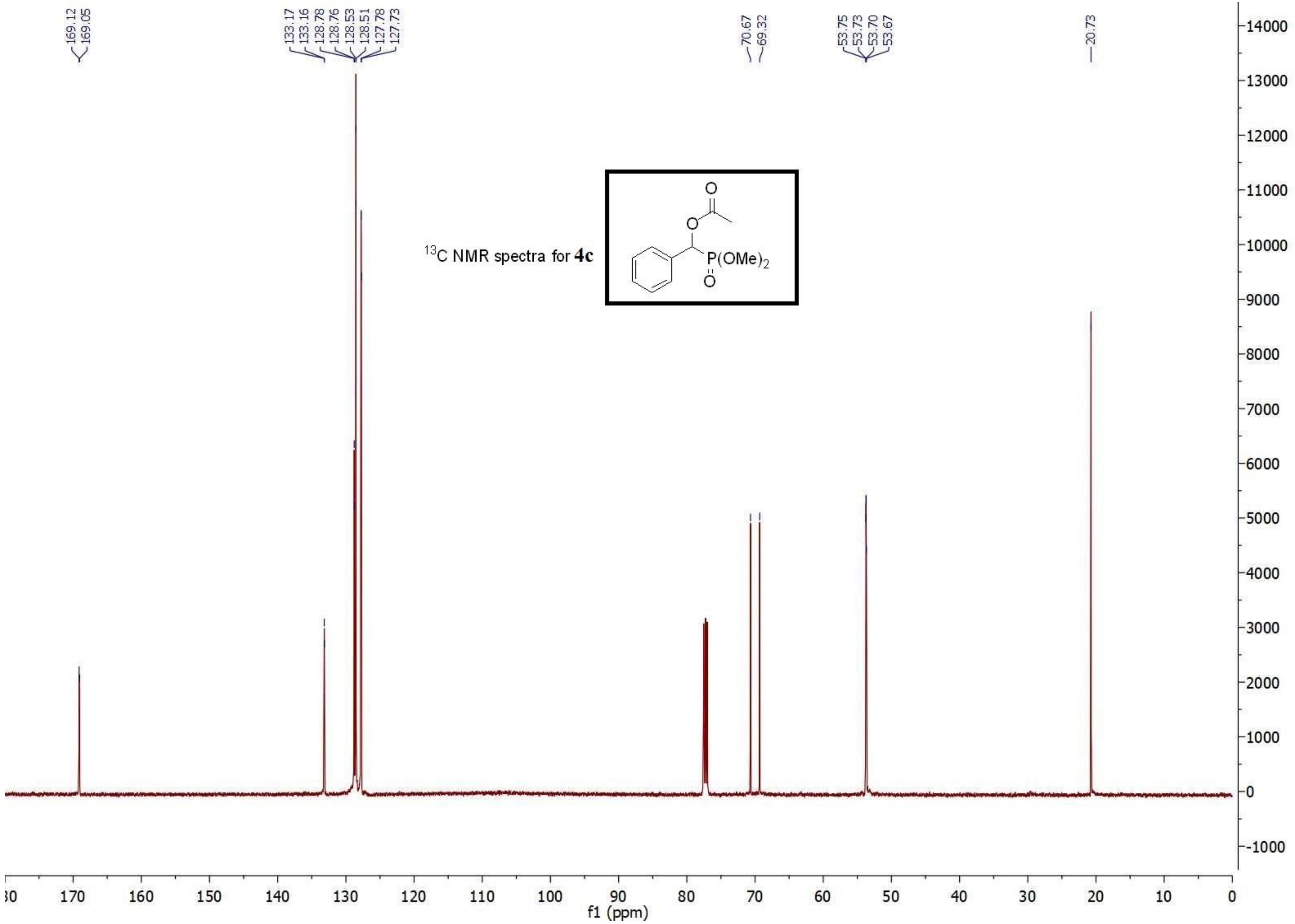

169.12
169.05
133.17
133.16
128.78
128.76
128.53
128.51
127.78
127.73
70.67
69.32
53.75
53.73
53.70
53.67
20.73
^{13}C NMR spectra for **4c**
O
O
P(OMe)$_2$
O
14000
13000
12000
11000
10000
9000
8000
7000
6000
5000
4000
3000
2000
1000
0
-1000
30 170 160 150 140 130 120 110 100 90 80 70 60 50 40 30 20 10 0
f1 (ppm)

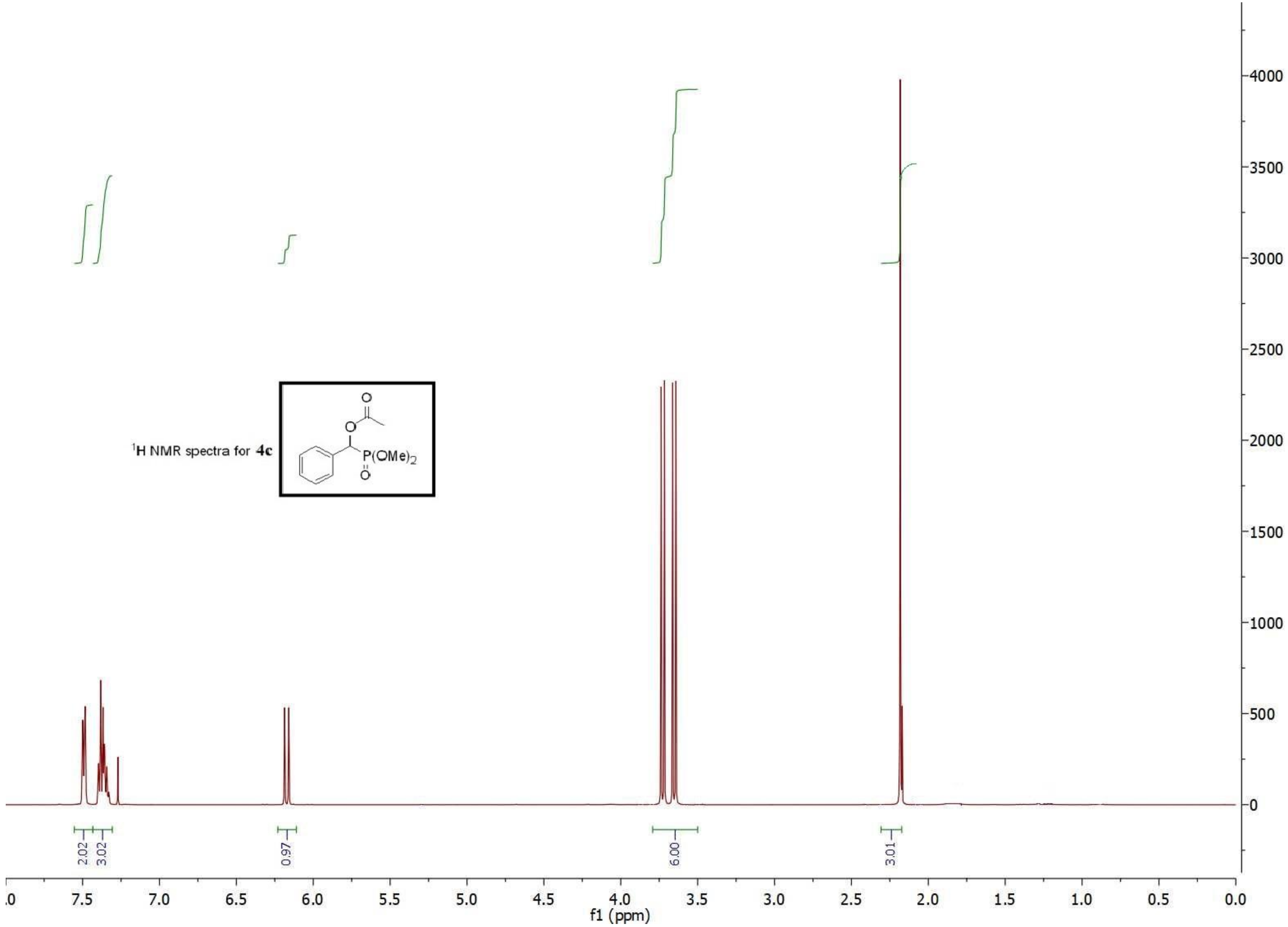

¹H NMR spectra for 4c
P(OMe)2
2.02
3.02
0.97
6.00
3.01
f1 (ppm)

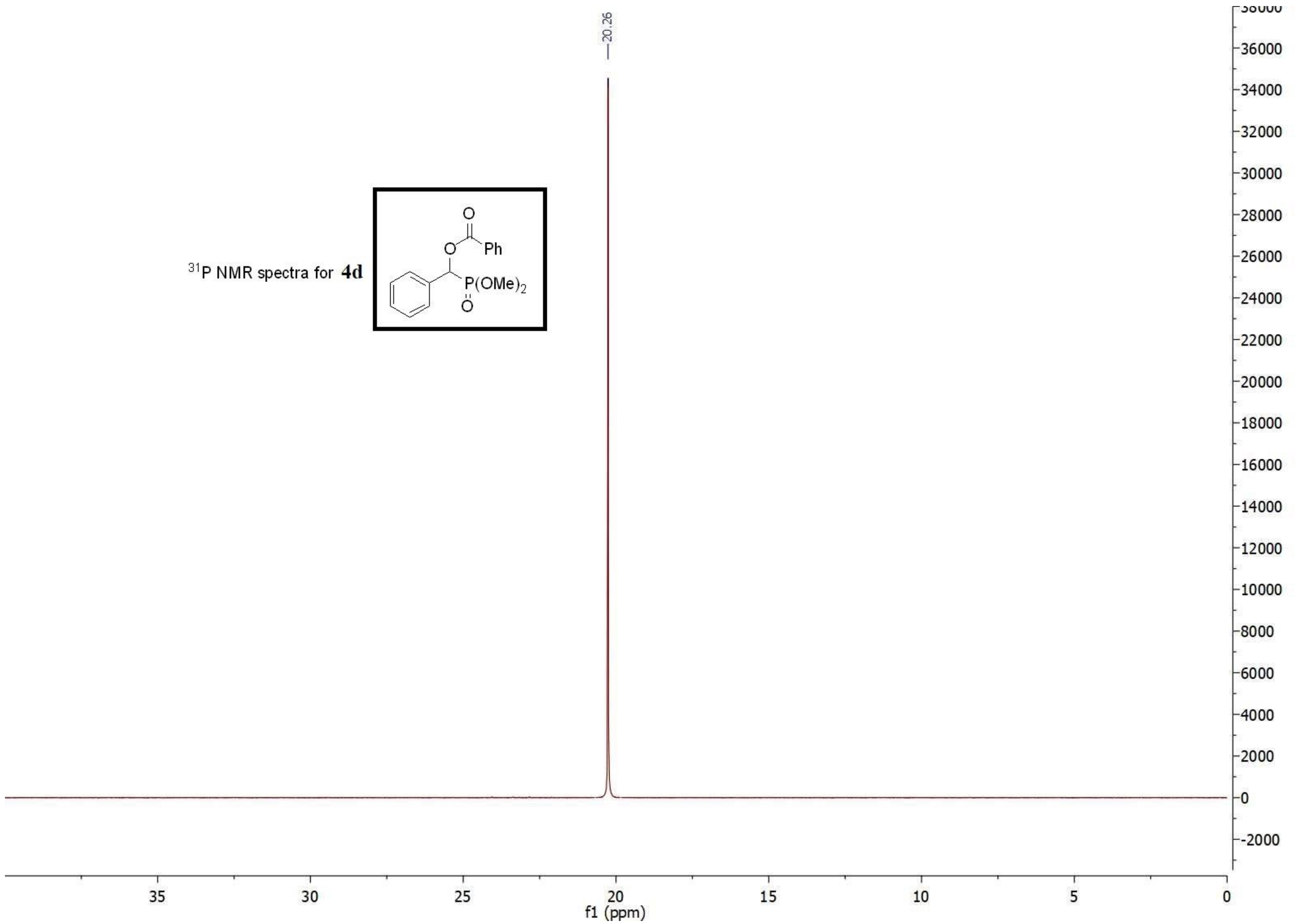

20.26
31P NMR spectra for 4d
O
O
Ph
P(OMe)2
O
f1 (ppm)

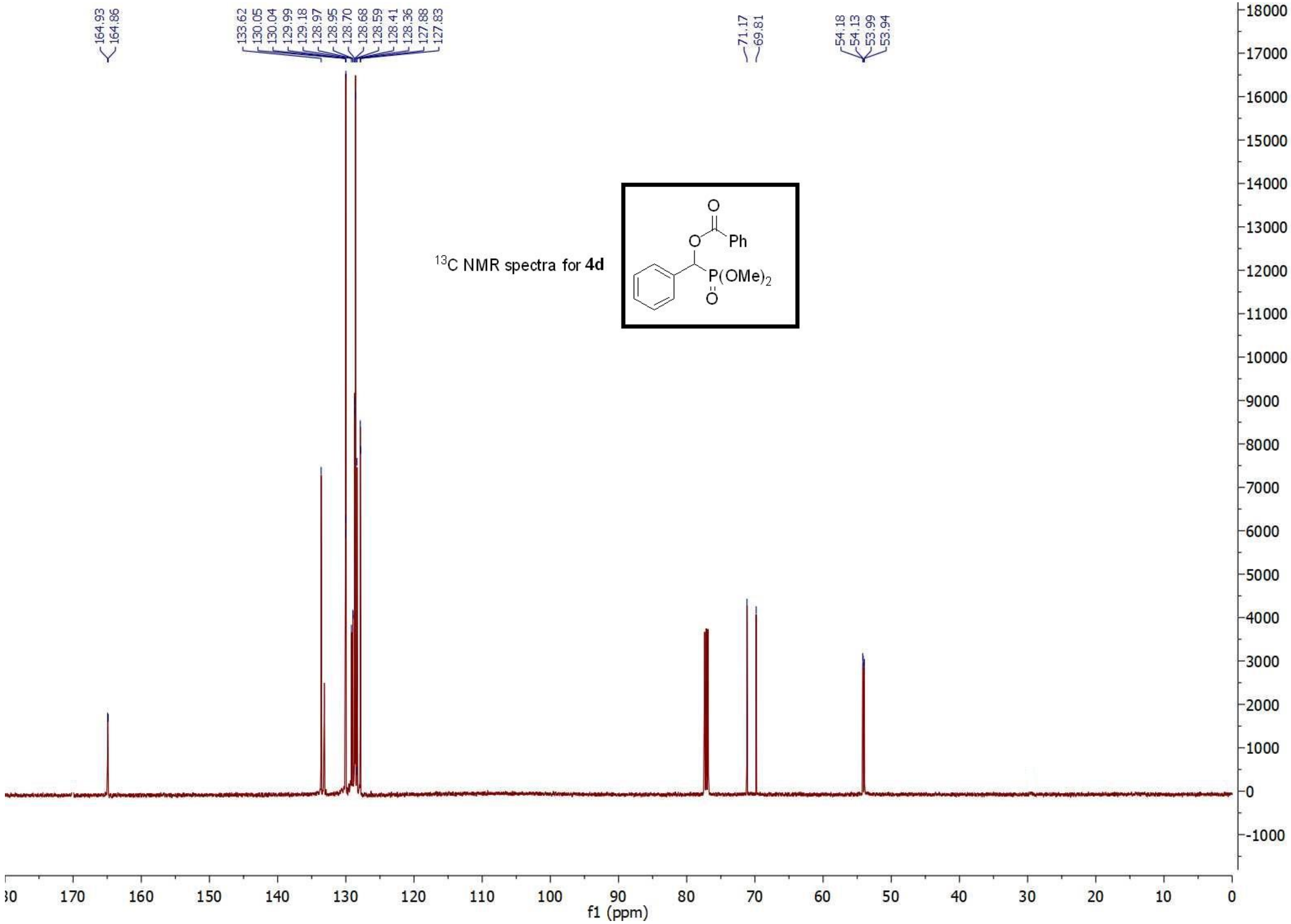

^{13}C NMR spectra for **4d**

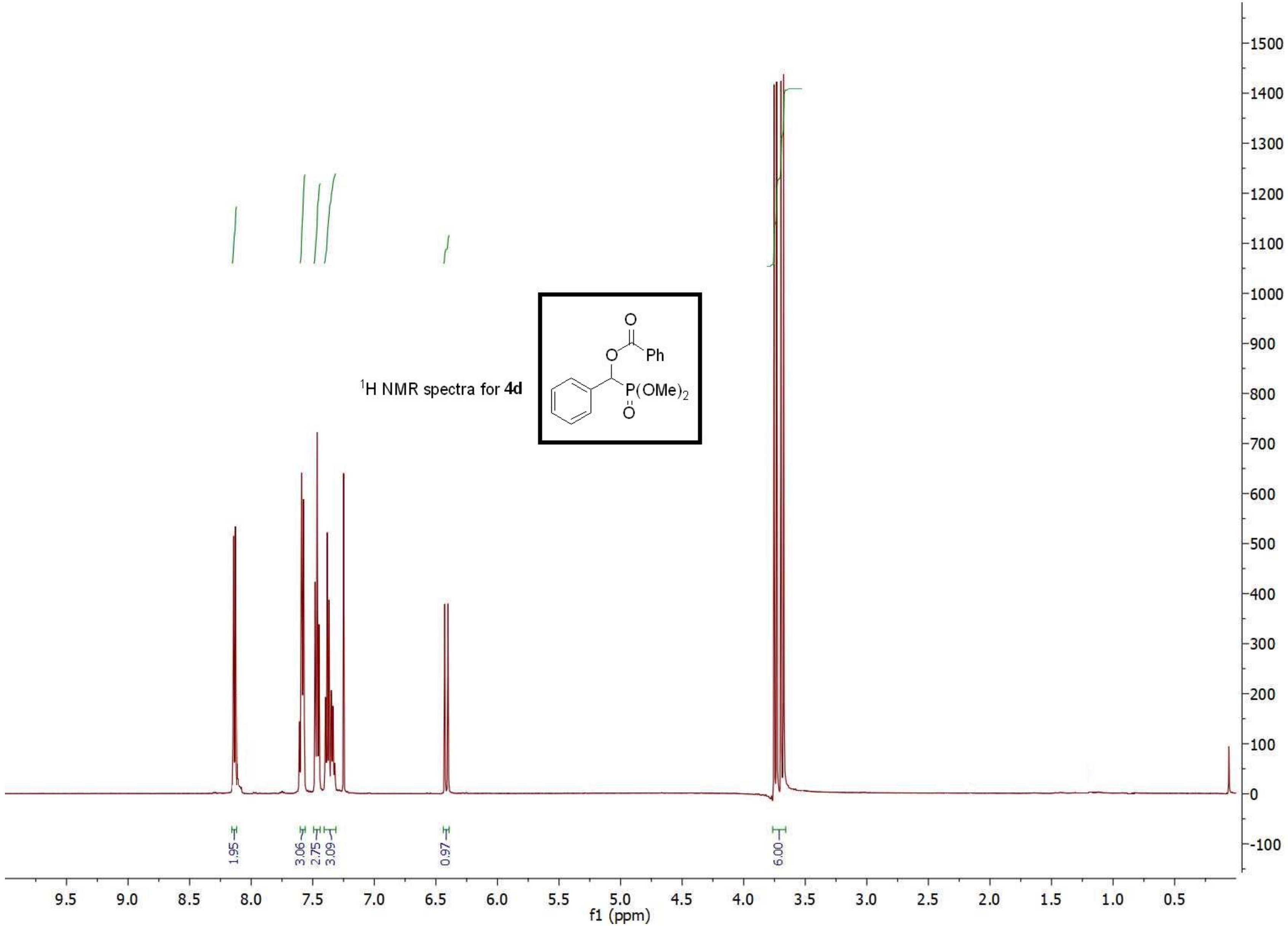

1H NMR spectra for 4d
O
O
Ph
P(OMe)2
O
1500
1400
1300
1200
1100
1000
900
800
700
600
500
400
300
200
100
0
-100
1.95
3.06
2.75
3.09
0.97
6.00
9.5
9.0
8.5
8.0
7.5
7.0
6.5
6.0
5.5
5.0
4.5
4.0
3.5
3.0
2.5
2.0
1.5
1.0
0.5
f1 (ppm)
